# An ecotoxicological view on neurotoxicity assessment

**DOI:** 10.1186/s12302-018-0173-x

**Published:** 2018-12-14

**Authors:** J. B. Legradi, C. Di Paolo, M. H. S. Kraak, H. G. van der Geest, E. L. Schymanski, A. J. Williams, M. M. L. Dingemans, R. Massei, W. Brack, X. Cousin, M.-L. Begout, R. van der Oost, A. Carion, V. Suarez-Ulloa, F. Silvestre, B. I. Escher, M. Engwall, G. Nilén, S. H. Keiter, D. Pollet, P. Waldmann, C. Kienle, I. Werner, A.-C. Haigis, D. Knapen, L. Vergauwen, M. Spehr, W. Schulz, W. Busch, D. Leuthold, S. Scholz, C. M. vom Berg, N. Basu, C. A. Murphy, A. Lampert, J. Kuckelkorn, T. Grummt, H. Hollert

**Affiliations:** 1 Institute for Environmental Research, Department of Ecosystem Analysis, ABBt–Aachen Biology and Biotechnology, RWTH Aachen University, Worringerweg 1, 52074 Aachen, Germany; 2 Environment and Health, VU University, 1081 HV Amsterdam, The Netherlands; 3 FAME-Freshwater and Marine Ecology, Institute for Biodiversity and Ecosystem Dynamics, University of Amsterdam, P.O. Box 94248, 1090 GE Amsterdam, The Netherlands; 4 Luxembourg Centre for Systems Biomedicine (LCSB), University of Luxembourg, 6 Avenue du Swing, 4367 Belvaux, Luxembourg; 5 National Center for Computational Toxicology, Office of Research and Development, U.S. Environmental Protection Agency, 109 T.W. Alexander Dr., Research Triangle Park, NC 27711 USA; 6 KWR Watercycle Research Institute, Groningenhaven 7, 3433 PE Nieuwegein, The Netherlands; 7 Department Effect-Directed Analysis, Helmholtz Centre for Environmental Research-UFZ, Permoserstr. 15, Leipzig, Germany; 8 Ifremer, UMR MARBEC, Laboratoire Adaptation et Adaptabilités des Animaux et des Systèmes, Route de Maguelone, 34250 Palavas-les-Flots, France; 9 INRA, UMR GABI, INRA, AgroParisTech, Domaine de Vilvert, Batiment 231, 78350 Jouy-en-Josas, France; 10 Ifremer, Laboratoire Ressources Halieutiques, Place Gaby Coll, 17137 L’Houmeau, France; 11 Department of Technology, Research and Engineering, Waternet Institute for the Urban Water Cycle, Amsterdam, The Netherlands; 12 Laboratory of Evolutionary and Adaptive Physiology, Institute of Life, Earth and Environment, University of Namur, 5000 Namur, Belgium; 13 Department of Cell Toxicology, Helmholtz Centre for Environmental Research-UFZ, Permoserstr. 15, 04318 Leipzig, Germany; 14 Eberhard Karls University Tübingen, Environmental Toxicology, Center for Applied Geosciences, 72074 Tübingen, Germany; 15 MTM Research Centre, School of Science and Technology, Örebro University, Fakultetsgatan 1, 70182 Örebro, Sweden; 16 Faculty of Chemical Engineering and Biotechnology, University of Applied Sciences Darmstadt, Stephanstrasse 7, 64295 Darmstadt, Germany; 17 Swiss Centre for Applied Ecotoxicology Eawag-EPFL, Überlandstrasse 133, 8600 Dübendorf, Switzerland; 18 Zebrafishlab, Veterinary Physiology and Biochemistry, University of Antwerp, Wilrijk, Belgium; 19 Institute for Biology II, Department of Chemosensation, RWTH Aachen University, Aachen, Germany; 20 Zweckverband Landeswasserversorgung, Langenau, Germany; 21 Department of Bioanalytical Ecotoxicology, UFZ–Helmholtz Centre for Environmental Research, Leipzig, Germany; 22 Department of Environmental Toxicology, Swiss Federal Institute of Aquatic Science and Technology, Eawag, Dübendorf, 8600 Switzerland; 23 Faculty of Agricultural and Environmental Sciences, McGill University, Montreal, Canada; 24 Department of Fisheries and Wildlife, Michigan State University, East Lansing, USA; 25 Institute of Physiology (Neurophysiology), Aachen, Germany; 26 Section Toxicology of Drinking Water and Swimming Pool Water, Federal Environment Agency (UBA), Heinrich-Heine-Str. 12, 08645 Bad Elster, Germany

**Keywords:** Eco-neurotoxicity, Neurotoxicity, EDA, REACH, AOP, Behaviour, Computational toxicity, Ecological, Species

## Abstract

The numbers of potential neurotoxicants in the environment are raising and pose a great risk for humans and the environment. Currently neurotoxicity assessment is mostly performed to predict and prevent harm to human populations. Despite all the efforts invested in the last years in developing novel in vitro or in silico test systems, in vivo tests with rodents are still the only accepted test for neurotoxicity risk assessment in Europe. Despite an increasing number of reports of species showing altered behaviour, neurotoxicity assessment for species in the environment is not required and therefore mostly not performed. Considering the increasing numbers of environmental contaminants with potential neurotoxic potential, eco-neurotoxicity should be also considered in risk assessment. In order to do so novel test systems are needed that can cope with species differences within ecosystems. In the field, online-biomonitoring systems using behavioural information could be used to detect neurotoxic effects and effect-directed analyses could be applied to identify the neurotoxicants causing the effect. Additionally, toxic pressure calculations in combination with mixture modelling could use environmental chemical monitoring data to predict adverse effects and prioritize pollutants for laboratory testing. Cheminformatics based on computational toxicological data from in vitro and in vivo studies could help to identify potential neurotoxicants. An array of in vitro assays covering different modes of action could be applied to screen compounds for neurotoxicity. The selection of in vitro assays could be guided by AOPs relevant for eco-neurotoxicity. In order to be able to perform risk assessment for eco-neurotoxicity, methods need to focus on the most sensitive species in an ecosystem. A test battery using species from different trophic levels might be the best approach. To implement eco-neurotoxicity assessment into European risk assessment, cheminformatics and in vitro screening tests could be used as first approach to identify eco-neurotoxic pollutants. In a second step, a small species test battery could be applied to assess the risks of ecosystems.

## Background

Neurotoxic pollutants are an emerging issue beyond human health because neurotoxicants causes potentially serious threats to vertebrate and invertebrate populations and ecosystems in general. Indeed, neurotoxic chemicals are suspected to produce changes in organism behaviour (e.g. mating behaviour, predator escape response and feeding behaviour), which can reduce an individual’s fitness, lead to population declines and ultimately have severe impacts on ecosystems [1]. Different classes of environmental contaminants, including metals and organic pollutants, were shown to affect the performance of complex behaviours in different fish species [2] and in wildlife [3, 4]. In wildlife, as in humans, early life stages are particularly sensitive to toxicant insults. Additionally, neurotoxic effects in early life stages might not be directly visible but lead to detrimental effects later in life. Besides, exposure to neurotoxic compounds can trigger epigenetic pathways which can underlie long-term effects as well as multi- or transgenerational effects [5, 6].

Typically, several thousand compounds are detectable in environmental samples, including synthetic and natural compounds and their transformation products [7]. However, knowledge regarding the neurotoxic potential of environmental contaminants in ecosystems is very limited, since the assessment of neurotoxicity is currently mostly focused on human exposure to individual chemicals. Known human neurotoxic or neuroactive compounds, such as pesticides, pharmaceuticals, and heavy metals, occur in the environment together with thousands of chemicals with unknown neurotoxic potential to different species and life stages. It has been estimated that up to 30% of all commercially used chemicals (~ 30,000 chemicals) may have neurotoxic potential [8]. Additionally, in a recent literature study looking at the known modes of action (MoA) of organic contaminants detected in freshwater monitoring studies, neurotoxicity was identified as the MoA linked to nearly 30% of all detected chemicals [9]. This shows the relevance of detecting neurotoxic compounds in the environment, increasing the demand for bioanalytical tools capable of identifying and possibly quantifying neurotoxic effects in organisms inhabiting contaminated ecosystems.

**Fig. 1:**
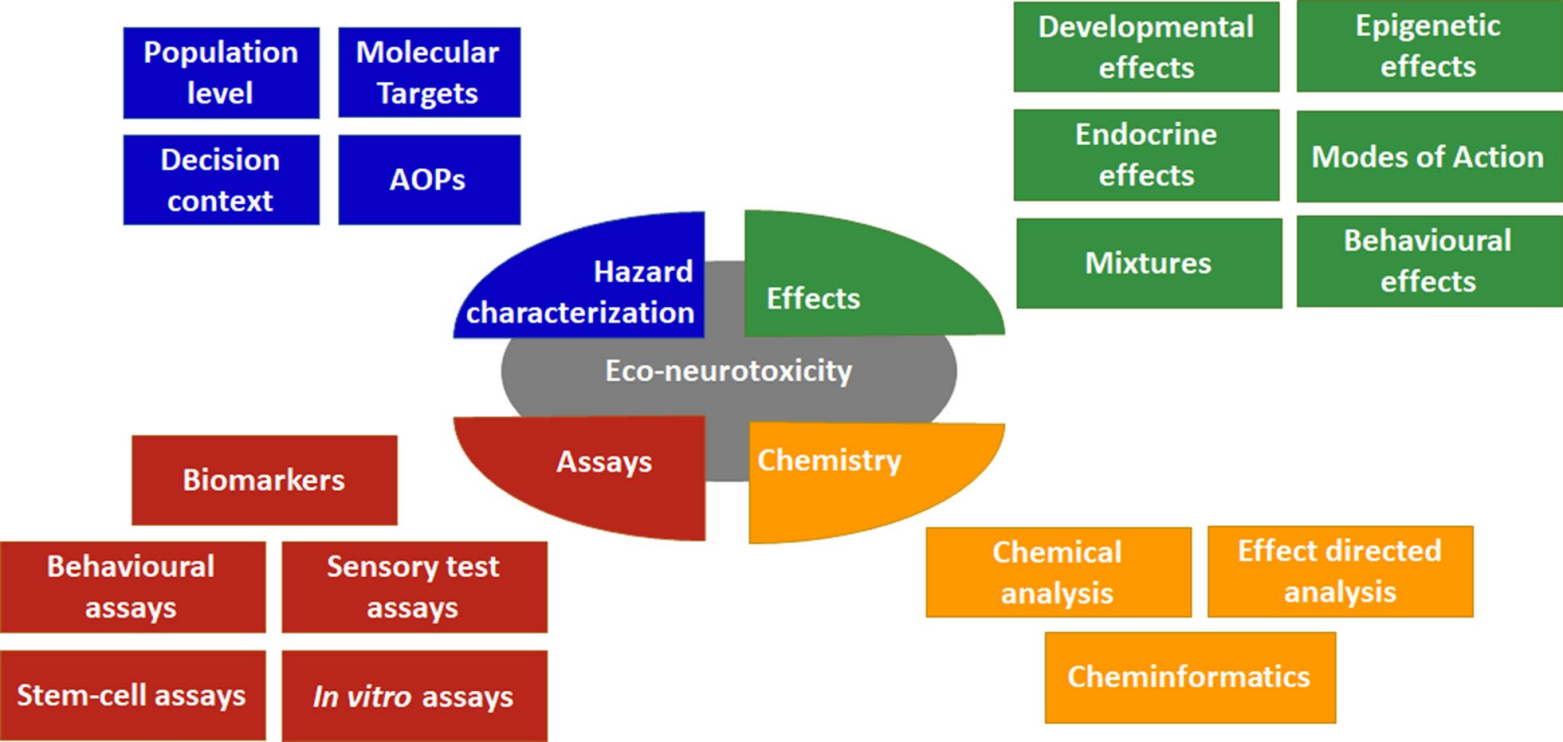
Key components of eco-neurotoxicity assessment

The aim of this article is to provide a critical overview of the state of the art of hazard characterization, effects, bioassays and chemical approaches regarding neurotoxicity in organisms as well as for ecosystems. This review will contribute a scientific perspective on the needs and future directions in neurotoxicity assessment for environmental protection (cf. Fig. 1).

## Environmental neurotoxicity versus eco-neurotoxicity

Neurotoxicity can be defined as the capacity of agents (chemical, biological, or physical) to cause adverse functional or structural changes in the nervous system [10]. Environmental neurotoxicity describes neurotoxicity caused by exposure to chemicals in the environment and commonly refers to human exposure and human neurotoxicity [10]. In contrast, we define ecological neurotoxicity (eco-neurotoxicity) as neurotoxicity resulting from exposure to environmental chemicals in species other than humans (e.g. fish, birds, invertebrates). It is important to distinguish between human and non-human neurotoxicity as the effects of exposure to compounds, both in terms of levels and pathways, as well as the structure and function of the nervous system itself, can differ widely between species.

## Current role of eco-neurotoxicology in risk assessment for regulation

### REACH/EU general food law

Within the current European chemical regulation, neurotoxicity is only assessed using in vivo test systems [11]. The EU legislation for industrial chemicals (REACH) assesses neurotoxicity only for compounds produced ≥ 10 tons/year. These compounds need to be tested with standard oral 28-day and 90-day toxicity studies in rodents. Clinical observations including motor activity, a functional observational battery and histopathological assessments of the spinal cord and sciatic nerve can be indicators of neurotoxicity. If these tests indicate neurotoxicity at levels below systemic toxicity, more detailed neurotoxicity tests are required (OECD technical guideline (TG) 424 to assess neurotoxicity and TG 426 to assess developmental neurotoxicity).

In terms of ecotoxicological impacts, current guidelines for neurotoxicity assessment in vertebrates focus on mammals and birds [12–16]. There is no regulatory guideline available to identify neurotoxic risks to other vertebrates or invertebrate animals. Furthermore, thus far there is no European regulatory framework for eco-neurotoxicity assessment.

Within risk assessment and risk management of eco-neurotoxic substances, pesticides are a substance class of special interest. Some pesticides kill pests via neurotoxic mechanisms. Neurotoxic actions on non-target species have been determined for several species and pesticides [17–22]. The European Food Safety Agency (EFSA) is responsible for the registration of pesticides and all other substances that can contact or occur in food and are not assessed under REACH. Until now, the active compounds in pesticides need to be assessed for potential neurotoxic effects in mammals using the same rodent studies as under REACH (TG 424 and TG 426) only if it is indicative from their intended MoA or other information, like chemical structure, that the substance could be neurotoxic [23]. Neurotoxic effects on non-target species in the environment are not assessed.

### Water Framework Directive

The European Water Framework Directive (WFD) aims to integrate biological and chemical information to obtain an overall insight into the quality of individual water bodies. According to the WFD, the chemical status of a water body is determined by analysing the concentrations of 45 priority substances, which are not selected based on their potential neurotoxicity. A good chemical status is defined by concentrations of all of these substances below the annual average and maximum allowable Environmental Quality Standards (AA- and MAC-EQSs), which are defined to protect the environment and human health [24].

As a result, regular chemical monitoring of the water quality is almost exclusively performed by targeted chemical analysis of a limited set of (indicator) compounds. There are, however, some serious limitations related to the use of target chemical analyses of large volume samples for monitoring the overall chemical status of a water body. First, because only a limited number of target substances are analysed, the risk of other, non-priority and unknown substances in the aquatic environment remains unknown [25]. At present (August 2018), more than 142,000,000 substances are registered in SciFinder with the Chemical Abstracts Service, while there are over 140,000 substances that are produced over 1 ton/year listed in REACH. Some of those compounds might eventually end up in the environment. Second, it is obvious that chemicals do not occur alone in the environment, but as complex mixtures. While concentrations of individual chemicals can be below the lowest observed effect concentrations (LOEC) or detection limits, the entire mixture may still cause adverse effects [26]. Moreover, transformation products of micropollutants formed in the environment or by biological metabolism are not always known or registered and may be more toxic and persistent than the parent compounds [27]. These limitations may thus result in an incomplete assessment of chemical hazards and risks, e.g. [28], urging alternative approaches to be explored [29].

### German drinking water ordinance

There is an urgent need for quick assessments of substances with unknown toxicological potential to prevent possible harm for consumers by water suppliers and public health departments, who supervise the process. At this time, there is no explicit regulation for neurotoxicity in drinking water in countries like Germany. While the German Drinking Water Ordinance (TrinkwV 2018) [30] gives threshold values for some metals, e.g. lead, cadmium, arsenic, with a known neurotoxic potential [31], no specific endpoints or proposals for a testing strategy are given for neurotoxicity.

The health-related indicator value (HRIV; in German: Gesundheitsbasierter Orientierungswert, GOW) concept provides a temporary value for toxicologically unknown single substances detected in drinking water systems. This hierarchically built concept is based on a precautionary in vitro approach with endpoints related to genotoxicity, neurotoxicity, endocrine disrupting effects and (sub-)chronic effects [32]. In a first step, several cell-based assays are used to detect effects of water concentrates or individual chemicals on basic parameters like apoptosis, necrosis and oxidative stress in HepG2 liver cells, Jurkat and U-937 blood cells. In a second step, organ-specific effects are compared between SH-SY5Y nerve cells and HepG2 liver cells using RTCA™ and Caspase assay. Finally, neurotoxic effects like neural differentiation of SH SY5Y cells are measured. Therefore, this concept can be used for high-throughput screening with the first and second test level and for determining neurotoxicity-effect concentrations in the third assay step. Furthermore, this approach can be applied to compare chemicals or exposure situations, although other neurotoxic mechanisms may remain obscured. The current approach could be extended also for eco-neurotoxicity assessment.

## Developmental eco-neurotoxicity

Developmental neurotoxicity (DNT) is particularly concerned with the effects of toxicants on the developing nervous system of organisms. The developing brain and nervous system is supposed to be more sensitive to toxic effects than the mature brain and nervous system [33]. Such studies must consider the temporal and regional occurrence of critical developmental processes of the nervous system, and the fact that early life exposure can lead to long-lasting or delayed neurotoxic effects [33].

Despite particular concern, the availability of information regarding developmental neurotoxicity of chemicals is very limited, even for humans. In a systematic literature review considering the neurotoxic potential of industrial chemicals to human populations, Grandjean and Landrigan identified 201 proven human neurotoxicants [34] and, moreover, they estimate that there are over 1000 compounds which were neurotoxic in laboratory animals, respectively [21]. Five of the 201 chemicals identified as human neurotoxicants were also classified as developmental neurotoxicants, while the other compounds could not be classified due to lack of experimental data [34]. Such low numbers demonstrate a clear lack of developmental neurotoxicity assessment studies. Additionally, a 2009 report indicated that only around 110 chemicals had been tested for potential human developmental neurotoxicity following respective OECD or US-EPA guidelines [35, 36]. As a consequence, there is a demand for time and cost-efficient testing methods capable of evaluating large numbers of chemicals for developmental neurotoxicity. Such methods may include in vitro and in silico tools as well as in vivo studies with alternative model species such as zebrafish (*Danio rerio*) [36]. Based on the 2009 reports [35, 36], an international collaboration was started led by Prof. E. Fritsche (IUF) with the goal to assemble a developmental neurotoxicity (DNT) testing battery for regulatory purposes. The in vitro testing battery will cover a variety of neurodevelopmental key events, distinct brain cell types and will investigate over 100 potential developmental neurotoxicants [37].

Developmental eco-neurotoxicity not only has to deal with similar challenges as for human neurotoxic investigations, such as complex temporal toxicity profiles due to different sensitivities of developmental stages combined with diverse target site susceptibility due to the complexity of the nervous system (depending on the species of interest), but in addition must consider the ecotoxicological perspective [38]. As a result, eco-neurotoxicity studies must aim to focus on protecting the most sensitive organisms and respective developmental stages among the multitude of different species in the environment.

In this sense, it is of great advantage that developmental eco-neurotoxicity can benefit from the knowledge obtained with model organisms such as the fish species medaka and zebrafish. Such fish models are used in both (developmental) neurotoxicity as well as in ecotoxicological studies, with investigations considering the involved MoAs and mechanisms of toxicity. For instance, changes in protein expression and whole mount antibody staining in medaka early life stages have been proposed as methodological approaches to characterize neurotoxic effects and respective mechanisms involved [39]. Zebrafish early life stages have been used as model organisms in a screening protocol to investigate environmental neurotoxicants considering various nervous system endpoints [40] and were proposed as systems toxicology models to support the identification of pathways of developmental neurotoxicity [41]. There are similar approaches with invertebrates, for example the characterization of sea urchins, which use neurotransmitters as embryonic growth regulatory signals, as model organisms for developmental neurotoxicity testing [42, 43]. Behavioural screening systems developed for zebrafish have also been applied for invertebrates like flat- and roundworms [44]. The nematode, *Caenorhabditis elegans*, is already a commonly used model organism for developmental biology and recently emerged as model organism for human neurotoxicity studies [45]. Their size makes them ideally suited for high-throughput behavioural screening approaches, whereas their well-known neurophysiology can be used to identify and study neurotoxic mechanisms. Although mostly used for human studies so far, flat- and roundworms could be easily used for environmental studies. There is also growing interest in using avian models, particularly the use of in ovo egg injection methods in which developmental exposures can be carefully controlled and linked with a range of structural and functional outcomes in the hatchling and later life stages [46, 47].

In the long run, developmental eco-neurotoxicity should integrate the outcomes of experimental investigations utilizing such ecotoxicologically relevant organisms with data from in vitro and in silico predictive models, in a similar way as proposed for humans [37]. In order to be successful, predictive developmental eco-neurotoxicity should consider the diverse mechanisms and MoAs involved, as well as their variation across species and toxicants, as already suggested for predictive ecotoxicology in support of ecological risk assessment [48, 49].

## Epigenetics in eco-neurotoxicity

Epigenetics can be defined as the study of changes in gene expression that occur without changes in the DNA sequence, and which may be heritable. Inheritance is understood in two different ways; mitotic inheritance (i.e. from cell-to-cell through cell division) and meiotic inheritance (i.e. from one organism to its offspring through reproduction) [50]. Three main epigenetic mechanisms are generally described: DNA methylation, histone modifications and non-coding RNA [51, 52]. Cell-to-cell inheritance involves the maintenance of epigenetic marks during the life of the individual, offering very interesting hypotheses for delayed effects of exposure to toxicants in early stages of life. On the other hand, transgenerational inheritance of epigenetic marks could explain how specific traits that were induced by exposure to toxicants can be observed in offspring that itself is not directly exposed [53].

DNA methylation is the most studied epigenetic modification and consists in the methylation of cytosine nucleotides in the genome by DNA methyltransferase (DNMTs). One particularity of DNA methylation is that it can be depleted and replaced again during epigenetic reprogramming events to set up cell- and tissue-specific gene expression [52, 54]. More precisely, DNA methylation patterns are reprogrammed across the whole genome in early embryos and primordial germ cells. This is well known in the case of mammals [55, 56], but it has also been observed in flowering plants and other animals such as fish [57–59]. This process is essential for a normal development of the animal brain as it modulates the expression of neural genes during specific developmental time periods, but it may also represent a particularly vulnerable period for an exposure to toxicants [60, 61]. Consequently, an early life stage exposure to neurotoxicants may thus impact the later or adult phenotype by interfering with reprogramming, leading to negative consequences on the development of the central nervous system (CNS) [6, 62].

The exact MoAs of neurotoxic compounds on the epigenome are almost completely unknown. Neurotoxic effects of pollutants can be channelled by oxidative stress, mainly interfering with the ability of DNMTs to link and interact with DNA [5, 6]. Similarly, transient exposure to chemical compounds such as bisphenol A or valproic acid in the womb can alter DNA methylation and histone deacetylation processes, which has been linked to persistent consequences such as defective brain development and memory loss in later stages of life [63]. Parallel work in metals demonstrates that epigenetics may be a critical pathway for metal-induced neurotoxicity as a result of Fe, As or Cd exposure [6]. Impairment of human and animal behaviour following either pre- or post-natal exposure to neurotoxicants has been recorded and linked with neurodegenerative diseases in adults [64–67]. However, the role of epigenetics in the development of these degenerative processes requires further research.

Beyond the organism’s lifetime, transgenerational epigenetic inheritance (TEI) may have critical implications for populations and species. The evaluation of a potential transmission of environmentally induced epigenetic modifications has been highlighted as a necessary field of research in ecological risk assessment [68]. Here, a clear distinction between intergenerational and transgenerational inheritance is required. The first involves a direct exposure of the germ cells that will later constitute the next generation, and the latter involves an indirect transmission of non-genetic information from one generation to another [69]. Evidence of TEI underpinning neurotoxic effects in multiple generations is very rare. A recent study by Knecht et al. reported transgenerational behavioural effects of benzo[a]pyrene on zebrafish exposed during development (hyper locomotor activity, hyper-avoidance behaviour) [70]. The same study also showed that global DNA methylation was decreased, as well as DNMT expression. Similarly, Carvan et al. showed a correlation between developmental induced transgenerational inheritance of abnormal behaviour in zebrafish exposed to methylmercury, and sperm epimutations in the second filial (F2) generation [71]. In another example, exposure of one generation of zebrafish to a complex mixture of PCBs and PBDEs simultaneously triggered changes in DNMTs expression and behaviour in larvae and/or adults in up to four non-exposed offspring generations [72].

The current understanding of epigenetics is strongly biased towards the use of laboratory animals such as mice and rats, which limits its applicability to eco-neurotoxicology. Nonetheless, the observed effects of neurotoxicity of some compounds in humans and laboratory animals can be transposed to wildlife: birds, terrestrial mammals or marine and freshwater organisms [73– 77]. For example, in a comparative study DNMT activity and DNA methylation were measured in brain tissues from methylmercury-exposed mink (mammal), chicken (bird), and yellow perch (fish), thus showcasing how relevant epigenetic measures can be incorporated into laboratory-based studies on ecologically relevant species [78]. Altered DNA methylation has also been shown in Daphnia exposed to toxicants [79], highlighting that epigenetic effects are not only occurring in vertebrates. Behaviour mediates the interaction between the organism and its environment, e.g. helping organisms adapt to new environmental conditions [80]. Appropriate behaviour is crucial for organisms to survive. Neurotoxicants in air, water and/or soil could affect the CNS and the behaviour of organisms and lead to changes in ecology, particularly if the consequences of exposure to neurotoxicants can be transmitted to following generations. Epigenetics research may hold the key to understand the mechanisms of transmission of such environmental information, potentially playing a role in processes of rapid adaptation [81]. This aspect of eco-neurotoxicity requires further investigation by the scientific community to improve the understanding of the molecular underpinnings of eco-neurotoxicity and its long-term consequences on ecosystems.

## Endocrine eco-neurotoxicity

Accurate spatial and temporal hormone signalling is required for correct neuronal development. Consequently, chemicals disrupting the hormone (endocrine) signalling during neurogenesis may cause severe, irreversible cognitive defects in exposed organisms [82]. For example, perturbation of the thyroid system was associated with motor and mental disorders in rats, apes and humans and the emergence of diseases such as attention-deficit hyperactivity disorder/syndrome [83–86]. This is of concern for regulatory authorities since endocrine active compounds are ubiquitous in the environment [87, 88]. Hence, an increasing number of researchers are investigating the link between endocrine disruption and neurotoxicity [82, 85, 89–93]. Several studies, especially with fish, show an impact of endocrine disrupting chemicals (EDCs) on behaviour, which is thought to be a representative endpoint for neurotoxicity [94– 99]. Evidence also exists for quails, tadpoles and a few invertebrate species, suggesting endocrine developmental neurotoxicity after exposure to EDCs. However, further research is needed to reveal possible links [91, 100–103].

While mechanisms causing developmental neurotoxicity remain unknown [92, 104], endocrine developmental neurotoxicity is of concern for the environment since hormone systems are conserved within animal taxa [105]. Adverse effects observed in the laboratory may thus occur in wildlife [82, 87]. To avoid effects on ecosystems and perform reliable environmental risk assessment, research needs to understand the mechanisms evoking neurotoxicity [93]. In addition, mixture effects, spatial and temporal exposure scenarios and community structures need to be considered [106–108].

## Neurotransmitter system related modes of action of eco-neurotoxicity

One of the MoA relevant for eco-neurotoxicity are disturbances in electric signal transduction and inhibition of chemical signal transduction, mainly through interference with the neurotransmitters [109]. Examples include the inhibition of the degradation of acetylcholine by blocking the enzyme acethylcholinesterase (AChE) in the excitatory synapses or by inhibition of the GABA (g-aminobutyric acid) ρreceptor in the inhibitory synapses [110].

Environmental pollutants such as DDT bind to open sodium channels in neurons, which prevents closing of the channels and leads to over-excitation [111]. Pyrethroids, such as permethrin, increase the time of opening of the sodium channels, leading to similar symptoms [112]. Lindane and cyclodiene insecticides block GABA-mediated chloride channels [113]. Organophosphate insecticides bind to AChE and hence prevent the degradation of acetylcholine, leading also to overexcitation and severe toxic symptoms, when over 50% of the AChE receptors are blocked. Neonicotinoids (e.g. imidacloprid) bind to the nicotinic acetylcholine receptors (nAChR), and their binding is irreversible but the potency is much higher on insect nAChR than on the corresponding mammalian receptors [114]. However, they are dangerous to non-target insects like bees and have been associated with a decline in the bee population [115]. Phenyl-pyrazols such as fipronil bind to GABA receptors and the selectivity for insects over mammals is also caused by a higher binding affinity [116].

**Fig. 2:**
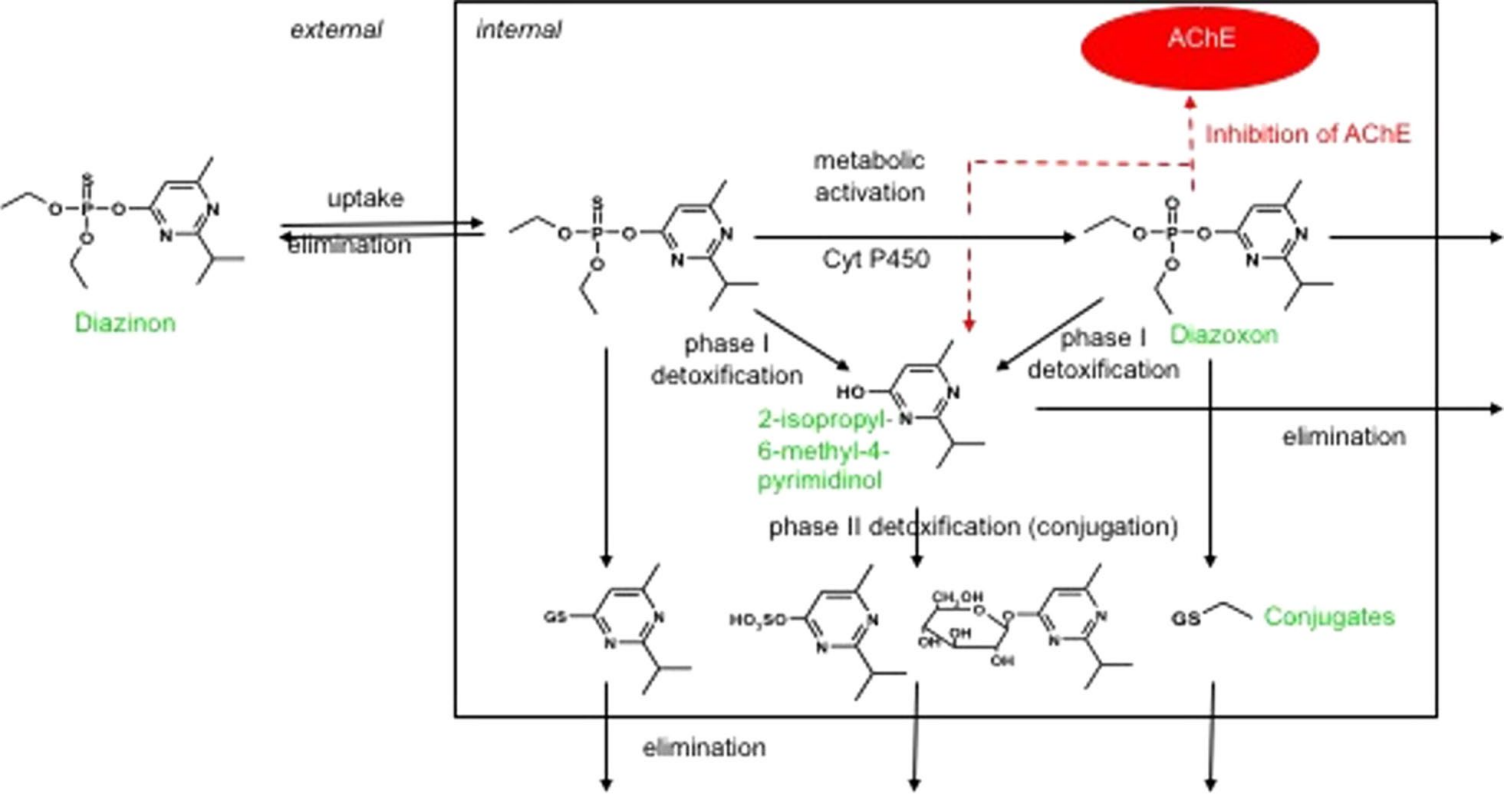
Mechanism of action of an AChE inhibitor on the example of the insecticide diazinon (simplified from [117]). After oxidation catalysed by cytochrome p450 monooxygenases, Diazinon is metabolized into Diazoxon which can inhibit acetylcholinesterase. Via further phase 1 and phase 2 metabolization steps the molecule is eliminated

Most organophosphate insecticides are thio-phosphoesters that require oxidation prior to causing inhibition of AChE as is illustrated by diazinon in Fig. 2. The oxidation catalyzed by cytochrome p450 monooxygenases transforms diazinon to diazoxon, which binds to the esterase site on the AChE by releasing the pyrimidinol species as a leaving group (in this example 2-isopropyl-6-methyl-4-pyrimidinol). The remaining AChE-phosphoester complex is then further hydrolysed, leading to a so-called ageing (irreversible binding) of the inhibitor-enzyme complex. AChE inhibitors that do not have a good secondary leaving group are reversible inhibitors as they do not age and can be released again from the complex. In contrast the aged complex is fairly stable, and recovery is mainly due to new formation of AChE. In parallel to activation and inhibition, both diazinon and diazoxon can be detoxified by carboxylesterases and the resulting pyrimidinol can be conjugated prior to elimination.

As detoxification is the dominant pathway in mammals and oxidation is the dominant pathway in invertebrates, organophosphate insecticides are typically more toxic to invertebrates [117] than to vertebrates [118]. Differences in species sensitivity have been explained by the interplay between activation by oxidation and detoxification. The fish carp was found to be less sensitive than other fish species (trout, guppy, zebrafish) despite a more sensitive AChE site because it had the most active detoxifying enzymes [119]. *Daphnia magna* was found to be more sensitive to diazinon than *Gammarus pulex*, partially due to a six times slower detoxification by carboxylesterases, which compensated for the twice faster oxidative activation in *G. pulex*, but mainly due to toxicodynamic differences as observed by applying a toxicokinetic–toxicodynamic (TK–TD) model to survival data for *G. pulex* and *D. magna* [120]. TK–TD models are especially suitable to investigate the time-dependent effects and complex mechanism of AChE inhibitors and allow estimation whether toxicokinetic or toxicodynamic parameters determine the overall effect [121]. Interestingly, for some organophosphate insecticides, the organism recovery is the rate-limiting step of toxicity [121]. For an uncoupler such as pentachlorophenol, the TK depuration and the TD organism recovery in *G. pulex* are 2 and 3 days, respectively; hence, the organism has basically recovered when it has eliminated the chemicals. However, this is different for carbaryl, chlorpyrifos and diazinon with a TK depuration of 1.4–11 days but a much longer organism recovery in the range of 15–28 days [121]. Hence, the sequence of exposure matters when mixtures are investigated over a time period [122].

Such detailed studies about the uptake, metabolism and excretion of neurotoxicants are extremely important as they help to link exposure to effects which is necessary to properly assess eco-neurotoxicity for different species.

## In vitro eco-neurotoxicity approaches

As mentioned above, the research field of neurotoxicology is mainly focused on the potential effects of chemicals on development, structure and function of the human nervous system. There is general agreement within the field of toxicology that state-of-the-art toxicity testing includes tiered testing strategies that focus on the pathways that are critical for adequate functioning of cells, organs, and organisms, using different testing strategies to collect information on exposure and toxicity. This includes an emphasis on non-animal models, including integrated genomic and proteomic analyses of chemical-induced effects. This principle is embraced in the neurotoxicology research field and many efforts are focusing on developing and optimizing non-animal test systems to model such effects by increasing their sensitivity and specificity [123].

Non-animal test systems to test neurotoxicity include small intact organisms (zebrafish embryos, *C. elegans*), brain slices, cell lines, primary cell models and stem-cell derived models as well as assays assessing the inhibition of the bare enzyme (e.g. in the case of AChE inhibition assays) [124]. Different types of model systems have their own advantages and disadvantages related to multicellular complexity, ease of culture, variability between cultures, possibilities with regard to differentiation or genetic modification, species and costs. Test systems with non-mammalian test organisms [125, 126] may include ecotoxicologically relevant species, or methods to study the neurotoxicity endpoints may be modified to enable an application for other species.

Many neurotoxic mechanisms can be studied in vitro using biochemical and morphological endpoints that have the potential for medium-to-high-throughput testing. Parameters to investigate neuronal network functionality include network formation, action potential generation, calcium homeostasis, synaptic transmission, and synaptic plasticity. However, these assays were developed with the aim of studying effects of chemicals on the human (mammalian) nervous system and not on wildlife. Nevertheless, if the goal is to investigate whether neuroactive chemicals or chemicals with neurodevelopmental toxicity potential are present in the aquatic environment, these in vitro cell systems may also be suitable for water quality monitoring. Both ecotoxicological model species and in vitro bioassays for molecular mechanisms are included in the Smart Integrated Monitoring (SIMONI) framework for water quality monitoring [127].

Innovative experimental approaches are available to investigate effects on neuronal function, such as optical and electrophysiological measurements of intra- and intercellular signalling (calcium signalling, neurotransmitter release and post-synaptic receptor function) in cell models to measurements of spontaneous activity or network activity in neuronal networks using multi-electrode arrays (MEAs) [124]. Chemical-induced changes in network function measured in a MEA system may be due to changes in electrical activity as well as in the release or reception of intercellular signals. MEA systems thus provide an integrated, but not pathway specific, measure for effects on neurotransmission. Efforts are ongoing to increase throughput by using multi-well MEA systems.

Primary (rodent) cell cultures can be used for routine neurotoxicity testing [128], and efforts are ongoing to increase throughput capacity of functional neuronal networks of (human) embryonic stem cells and neural/neuronal progenitor cells [129, 130]. The generation of large amounts of data, either by testing many samples using high-throughput approaches or by generating high density data (e.g. using MEAs or optical recordings), requires ample data storage. The resulting challenges associated with data analysis require the implementation of chem/bioinformatics approaches.

If the aim of effect-based monitoring is to study the impact of neuroactive chemicals, or chemicals with neurodevelopmental toxicity potential, on ecologically relevant species, it is critical to consider (dis)similarities in brain development, structure and function between mammals and ecotoxicologically relevant species. Considerable underlying differences may exist between mammals and other taxa in sensitivity to neurotoxic chemicals [131]. For example, it is well known that, due to interspecies differences in kinetic parameters of AChE, insects are more sensitive to organophosphate insecticides than mammals [132]. Moreover, sensitivity to neurodevelopmental effects resulting from exposure to chemicals depends critically on the phase of mammalian brain development [33]. Specific sensitivity for neurotoxicity dependence on exposure timing may need to be investigated further using ecotoxicological model species. Additionally, neuronal network function depends critically on the presence of multiple cell types [133], including neurons, oligodendrocytes, microglia and astrocytes. The importance of multiple cell types for eco-neurotoxicology may depend on the endpoint of interest. It is thus required to identify the most relevant neuronal cell types and the impact of absence or presence of other cell types in the in vitro test system under consideration.

Due to the complexity of neurotoxic and neurodevelopmental mechanisms, and in view of potential differences between mammals and other species, it is recommended to develop a specific test set of chemicals for eco-neurotoxicity (including, for example, water relevant chemicals and model chemicals that affect relevant mechanisms) for (interlaboratory) studies to test candidate in vitro bioassays (e.g. based partly Aschner et al. [134]). Emerging techniques and innovations in neuroscience and neurotoxicity should be closely followed and assessed for their potential and applicability in ecotoxicological water quality monitoring.

### Stem cells in eco-neurotoxicity

In the area of neurotoxicity and developmental neurotoxicity testing, the use of pluri- and multipotent stem cells differentiating into diverse neural cell types as well as standardized methods for differentiation will lead to an improved understanding of chemically induced adverse reactions. In contrast to cell lines or primary cells, stem cells and their derivatives are neither genetically transformed nor easily lose their tissue characteristics. However, differentiation conditions need to be strictly controlled to prevent differences in cell characteristics between cultures, which calls for appropriate control conditions to be included in toxicological testing procedures with stem cells. Neural differentiation occurs early in development and the formation of glial cells and neurons can quite easily be mimicked in vitro [135–138]. Thus, stem cells facilitate high-throughput neurotoxicity testing on a wide range of neural cell types. In this context, human induced pluripotent stem cells (iPSCs) have the potential to play an important role in predicting human-specific neurotoxicity and DNT [139]. Stem cell derived neuronal and glial models allow MoA-based DNT testing [37]. In recent years, the number of studies using stem cells for neurotoxicity and DNT testing with a variety of endpoints increased considerably. Many representative studies investigating neurotoxicity or DNT of drugs using stem cells applied endpoints such as cell-specific cytotoxicity (apoptosis, necrosis), cell migration, intracellular Ca^2+^ levels, disordered differentiation, neurite and dendrite outgrowth, neural network formation and activity, as well as synaptogenesis and synaptic activity [140–145]. Furthermore, 3D models such as neurospheres are now available and provide an improved comparability to the in vivo situation. Progress on in vitro 3D brain models has also been achieved [146]. 3D brain organoids derived from pluripotent stem cells are promising experimental models for brain development, DNT testing and neurodegenerative disorders enabling mechanistic pathway and stage-specific studies [147]. Despite this good progress in neurotoxicity and DNT testing with stem cells, validated methods for several endpoints still have to be established.

Mechanisms employed in the in vitro stem cell neurotoxicity and DNT testing are in general highly conserved in evolution. Results based on highly conserved mechanisms can be transferred to a variety of species, including aquatic organisms.

By now, many protocols for generating specific neuronal populations of different areas of the brain and peripheral nervous system using human iPSCs are available, and their efficiency is continuously increasing [148]. To date a general problem is that the protocols generate a heterogenous population comprised of the specific neurons they aimed for and also other cells, which may have a different identity. This increases the variability of potential test readouts. Also, especially for peripheral neurons, very long maturation times are needed to generate mature neurons, and tests performed at an earlier time point may be disturbed by the immature cellular answer of some of the cells. Additionally, some protocols suffer from a low reproducibility and relatively high variability. Thus, many repetitions are need, which renders the potential tests time and cost intensive. Current research is focusing on improving these shortcomings, and one solution is already in use: differentiating iPSCs into neuronal precursor cells offers the possibility to freeze and store larger amounts of cells of one differentiation, which then can be defrosted for the tests. This has the advantage that several tests can be run with the cells that were generated at the same time, at least until the precursor state. In most protocols for neuronal differentiation, many cells are lost during the first few days, thus making it hard to predict the cell number resulting from a single differentiation, even though a constant number of iPS cells were used. Now, one can defrost neuronal precursor cells and use a specific number of these cells for the last steps of the differentiation protocols, during which cell loss is almost negligible. This approach allows for a better control over the total number of generated neurons, thus increasing the comparability between different sets of tests.

Using gene-editing and other genetic methods progenitor cells can, e.g. be transduced to stably express Ngn2 under a Tet-On Advanced transactivator, allowing differentiation to be switched on by adding tetracyclin [149].

Apart from mice and rats, which often serve as model systems for human diseases, iPSCs from other mammals, such as farm animals [150], pets such as dogs [151] or endangered wild animals such as felids or orangutans [152, 153] are reported and offer a whole new set of opportunities to study the impact of environmental toxicity on their physiology.

A drawback of iPSCs is that the cells lose their epigenetic signature during reprogramming. To overcome this problem, somatic cells were used to transdifferentiate into neurons, bypassing the iPSC-state. Although to date the yield of this method is low and protocols show high variability, this allows to study age-dependent effects and may be a worthy option for eco-neurotoxicity testing in the future (see, e.g. collection of papers here: [154]).

### Cell-free neurochemical methods

Cell-free neurochemical assays are simplified in vitro systems that may help evaluate the effects of a test chemical on neurobiochemical processes. Cell-free assays are performed with cell lysates, tissue homogenates or with purified membranes (but not with living cells) and give information about direct biochemical interactions between test molecule and biological targets like receptors. They form an important assay category within the US-EPA’s ToxCast program and show promise for use in ecotoxicology as discussed by Arini et al. [155]. A great advantage, for example, is that they are amenable for use from any species from which brain tissue can be obtained, with one study comparing responses across 20 species of fish, mammals and birds [156]. Besides studying chemicals, cell-free assays have also been used to screen extracts from real-world samples including pulp and paper mill effluents [157] and wastewater effluents [158].

## Sensory system tests in eco-neurotoxicity

Neurotoxic effects on sensory systems are mostly studies in vertebrates. Little is known about effects in invertebrates. Sensory structures receive information from the environment and transduce it into a signal recognizable to the nervous system. The information or stimuli can be of different modality including light, sound, smell, taste, pressure and temperature. Generally, receptive cells contain transmembrane receptors which undergo a conformational change upon stimulation. A signal transduction cascade leads to the opening of ion channels and concomitant membrane potential changes, thereby creating an action potential. Many behaviours like feeding, mating, predator avoidance, migration, social interaction and communication are crucially informed by sensory systems. Thus, their impairment can have severe impact on fitness and survival of an animal.

Environmental contaminants such as pharmaceuticals, pesticides and heavy metals have been shown to interfere with the sensory structures of different species including humans and fish, thereby creating deficiencies in sensation and behaviour. Behavioural output is an increasingly measured, ecologically relevant and very sensitive endpoint. To localize specific sensory impairments within the nervous system using behaviour is, however, challenging because behaviour is the integrated output of multisensory, neuroendocrine and neuromuscular signals, and tests are often not specific enough (e.g. impaired feeding might result from motor deficits, impaired olfaction, impaired vision or a combination thereof).

Generally, four techniques listed in Table 1 are applied to assess the different sensory systems like, e.g. olfaction, vision and mechanosensation (discussed below). While all of them have their advantages and drawbacks, there is no recommendation as to which one is the best. Rather, tests have to be tailored to the study purpose and a multidisciplinary integrated approach is necessary to fully understand neurotoxicity mechanisms [2].

**Table 1:** Techniques to test sensory system

	Sensitivity	Throughput	Specificity	Remarks
Electrophysiology	+++	+	+++	Link to behaviour often unclear, sophisticated preparations needed
Behaviour	++	+++	+	Multisensory input; depends on proper locomotor function; high ecological relevance
Anatomical changes	+	++	+++	Only apparent when sensory function already impaired
Molecular markers	+/+++*	+++	+/+++*	Good sensory toxicity markers are still rare

* Depending on marker

### Olfaction

In fish, the olfactory system is particularly vulnerable to neurotoxic contaminants because of the direct contact of olfactory sensory neurons with the surrounding water. Reduced or absent ability to smell (hyposmia or anosmia) have been shown to occur upon exposure to metals, pesticides and other contaminants like, e.g. surfactants [159]. The classical method to assess olfactory impairment is by electro-olfactography [160]. It assesses electrophysiological changes in olfactory sensory neurons by extracellular recordings. Olfactory behavioural tests include either attraction to food extract, avoidance of skin extract or attraction/avoidance of the chemical itself [159]. Notably, while the zebrafish olfactory system offers several experimental advantages to study sensory neurobiology in general and olfactory neurotoxicity in particular, there are a number of profound differences that might render translation of results from such studies to other species.

Among the major advantages of the zebrafish model are (i) identified ecologically relevant classes of natural odours such as amino acids and bile acids [161, 162]; (ii) cultivation of the adult zebrafish head ex vivo without anaesthesia, allowing neurophysiological measurements in the intact brain [163]; (iii) comparably small brain size that provides access to larger fractions of neurons by multiphoton microscopy [164]—a fact particularly true for the zebrafish olfactory system, which contains relatively few neurons and glomeruli [165]; and (iv) large detailed data at both the single neuron and population level that allows realistic mathematical simulations of circuit function [166]. Moreover, at first glance, the zebrafish olfactory system contains molecular and cellular constituents that appear similar in organization to the rodent olfactory system, thus, providing an attractive vertebrate model system to investigate the mechanisms underlying olfactory system development and function. However, the fish olfactory epithelium is of a “mixed” type, containing two major types of olfactory sensory neurons, i.e. ciliated and microvillous neurons. Both express distinct types of chemosensory receptors, project to different brain regions and likely mediate different behaviours [167]. Moreover, the three canonical zebrafish chemosensory gene families (*or*, *taar* and *olfC/V2r*) are somewhat unique and quite distinct in size and relative proportions to those of most tetrapods (indicative of the divergence of both lineages ~ 430 million years ago [168].

The mouse has become the most widely used model system in olfactory research based on established protocols for genetic manipulation. An important distinction between the olfactory systems in fish and mice is stimulus delivery. While the fish olfactory organs are exposed to pollutants and xenobiotics that are dissolved in water, rodent noses constantly sample volatile air-borne chemicals at minute concentrations. Thus, the range of potentially hazardous chemicals that water-living species like fish are naturally exposed to will be dramatically different from the repertoire of potential harmful compounds that land-living species like mice encounter (and vice versa). The olfactory system’s remarkable capacity to renew upon perturbation also needs to be taken into account [169]. The olfactory epithelium has extensive neurogenic and regenerative capacity in both rodents and humans that persists throughout adult life and is unmatched elsewhere in the nervous system [170]. Cells within the basal epithelial layer function as neuronal precursors, multipotent progenitors and/or stem cells. However, the niche signals that control the self-renewal and differentiation of these basal cells are not well understood [170]. This regenerative capacity will strongly impact eco-neurotoxicological assays that target the olfactory system. Accordingly, the system’s vulnerability will, at least to some extent, be compensated by adult neurogenesis.

Few neurotoxicological assays have been developed using mice. This is somewhat surprising given the large body of knowledge available, established animal care facilities, comparably short generation turnover and the large translational promise that rodent model systems offer. Thus, it appears likely that future eco-neurotoxicology assays will utilize a pipeline that spans cell-based in vitro experiments, high-throughput behavioural assays in zebrafish and other ecologically relevant species.

### Vision

About 3000 chemicals are toxic to the human eye and visual system [171]. Retinotoxic effects for organic solvents and metals have been described, not only for humans [172] but also for fish [173–176], for which most literature focuses on effects of methylmercury and ethanol. Moreover, the fish retina was affected upon herbicide [177, 178] and pesticide exposure [179] and was shown to accumulate cocaine [180]. Electrophysiological measurements of retinal function are called electroretinograms. They record the retinal sum field potential to a visual stimulus [181] and are applied in many species including fish [182]. Visual behaviour tests for fish are well developed, even to the point that different visual properties like motion detection, colour detection and discrimination, object recognition and visual acuity can be tested [183]. A popular assay is the measurement of optokinetic reflex, in which the animal is presented a moving grating which it follows with accurate eye movements. Impaired visual function results in reduced or absent eye movements [184, 185]. Although both techniques are widely established in zebrafish models for human ocular diseases [186], toxicant-induced impairments of visual function are only scarcely studied. Instead, retinotoxic effects are mostly assessed based on rather insensitive endpoints like histology or eye size (e.g. microphthalmia = smaller eyes).

### Mechanosensation

Hair cells are sensory cells of the vertebrate inner ear and the lateral line system of aquatic vertebrates. They transduce pressure changes in the surrounding medium into a neuronal signal as a result of deflection of their cilia, which leads to the opening of ion channels, enabling the detection of acoustic stimuli and hydrodynamic flow. Many drugs such as aminoglycoside antibiotics, platinum-based anti-cancer drugs, anti-malarics or non-steroidal anti-inflammatory drugs are known to induce ototoxicity in humans (see references in [187]), for the most part irreversible. In fish, some of these drugs equally cause ototoxicity and damage to the lateral line [188]. Moreover, metals such as copper, cadmium and others have been shown to cause hair cell death and deficits in behavioural responses in zebrafish [189, 190] and other fish [188]. Behavioural responses to acoustic stimuli [191–193], responsiveness to water motion [194] and rheotaxis (counter-flow swimming) [195, 196] have been measured to assess hair cell function. Moreover, vital dyes to stain hair cells have been widely used to assess the structure of lateral line hair cells in zebrafish [197]. Effects on the lateral line hair cells are one of the most promising sensory endpoints because of their great accessibility, amenability for staining’s and dyes and straightforward implication in rheotactic behaviour [198]. Another method to assess hearing abilities in fish is sound-evoked potential audiometry, which measures field potentials in response to an auditory stimulus using cutaneous electrodes [199].

In order to assess the full neurotoxic potential of environmental pollutants, a combination of tests and the assessment of multiple sensory systems are necessary to precisely localize effects within the nervous system [2, 200]. Future studies should strive to increase our mechanistic understanding of chemical neurotoxicity, which would help predicting eco-neurotoxicological effects. In this respect, model organisms such as the zebrafish are very helpful, because a large variety of genetic tools and genomic resources are available and many tests are already established for the analysis of human brain disorders [98], but they are not yet fully adopted for neurotoxicity testing. Additionally, emerging neuroscience techniques such as in vivo 2-photon calcium-imaging of neuronal activity [201–203] or optogenetics [204] might hold underexplored opportunities for the mechanistic dissection of complex neurotoxicological processes. Moreover, large-scale toxicity screenings using the zebrafish model has been implemented in the framework of ToxCast and Tox21 [205–208], but more efforts are needed to increase the specificity of tests for sensory neurotoxicity in larval zebrafish and implement them in a high-throughput manner in order to keep pace with toxicity testing of the vast number of newly registered chemicals.

## Biomarkers of eco-neurotoxicity

Biomarkers are defined as molecular, biochemical, cellular and physiological changes, caused by external stress factors. The two mostly discussed groups of biomarkers are: biomarkers of exposure that allow statements about the quality and/or quantity of exposure, whereas biomarkers of effect allow statements about effects and the health status of exposed organisms [209]. Classical examples for the first category are metallothioneins, which indicate metal contamination [210]. A typical biomarker of effect is the induction of stress proteins (heat shock proteins) [211] or a decrease of lysosomal stability [212]. With the latter two one can tell that the organism was exposed to environmental stressors, but it is not possible to tell, which stressor or contaminant exactly caused the observed effect [209]. Biomarkers can either be measured invasive/destructively, e.g. by determining enzyme inhibition in brain or whole-body homogenates or non-destructively, by determining the biomarkers of interest, e.g. in blood, mucus or skin samples [213, 214].

Biomarkers of eco-neurotoxicity include parameters reacting specifically to neurotoxic chemicals. The most well-known biomarker of effect for neurotoxicity is the measurement of AChE inhibition. This is the primary mechanism of action of organophosphate and carbamate insecticides. Enzyme activity is quantified in brain or whole-body homogenates of exposed organisms, and compared to reference inhibitors, as has been shown, e.g. for zebrafish embryos [215], fish [216] and *Daphnia magna* [217]. In addition, non-destructive measurement of cholinesterase is possible, e.g. by determining butyrylcholinesterase in blood serum [218, 219]. Alternatively, commercially available isolated AChE can be used to test chemicals and complex environmental mixtures such as water samples. The tests applied are typically based on the Ellman assay [220], which is a colorimetric assay that detects the hydrolysis of the substrate acetylthiocholine. Despite its wide application in water quality assessment, the assay using isolated AChE should be used with caution because concentrations of organic matter as low as 2 mg_C_/L, when present in solid-phase extracts of typical surface water, can act as non-specific inhibitor of AChE [221]. This can lead to an overestimation of insecticidal activity in ambient samples. The application of cholinesterase biomarkers for environmental monitoring has been reviewed by Mineau [222].

Other biomarkers of eco-neurotoxicity involve key neurotransmitter pathways and the measurement of corresponding enzyme inhibition or receptor activity, i.e. neurochemical biomarkers. Apart from AChE and the corresponding nicotinic and muscarinic receptors, activity of monoamine oxidase with dopamine or serotonin receptors, GABA transaminase with GABA(A) and GABA(B) receptors as well as glutamic acid decarboxylase and glutamine synthetase with the receptors NMDA, AMPA and Kainate can be determined. These biomarkers have been applied in studies on a variety of organisms such as worms, bivalves, fish, mammalian wildlife and birds (for review see [38]). For example, documented measurements have included a decrease in serotonin levels and an increase in monoamine oxidase levels in caged mussels in a river downstream of wastewater treatment plants effluent [223]. If mussels were exposed to primary treated and ozonated effluents in a flow-through experiment, GABA levels as well as the activities of several neuroactive enzymes (glutamic acid decarboxylase, monoamine oxidase and AChE) were reduced and levels of serotonin and dopamine increased [224]. With regard to fish, the application of biomarkers of eco-neurotoxicity has been reviewed in several publications [2, 225, 226]. In juvenile rainbow trout, altered brain neurotransmitter metabolism after exposure to β-naphthoflavone and benzo(a)pyrene resulted in impaired availability of serotonin at short term (after 3 h) and increased neuronal metabolic utilization of serotonin and dopamine after 24 and 72 h [227].

For any study, it has to be taken into account that the flexibility (plasticity) of the nervous system, as well as the age and developmental stage of the investigated organisms, may play an important role in the measured responses [38].

## Behavioural screening tests

For aquatic eco-neurotoxicological screenings, behavioural toxicity tests in small organisms may be of special interest. Behaviour is an understudied but sensitive and ecological relevant endpoint in ecotoxicity testing for all kinds of different species. Several studies reported effects on behaviour at concentrations orders of magnitudes below lethal concentrations [228, 229]. Behaviour is the integrated response of the conditions to which an organism is exposed. A variety of activities are used as behavioural endpoints to screen for effects of chemicals, for example avoidance, feeding and locomotion [1]. Some of these behavioural endpoints may be applicable to investigate rapid acute neurotoxic responses or effects of longer exposures with consequences that may have larger impact, such as neurodevelopmental effects. Such effects on behaviour may be caused by acute neurotoxic effects on neuronal functioning (inter- and intracellular signalling and neuronal network function to receive, conduct, and transmit signals via chemical or electrical synapses and relay information between specific brain regions for information processing as well as learning and memory formation) or to mechanisms related to neurodevelopmental processes (proliferation, migration, differentiation, formation of axons, and dendrites, synaptogenesis, network formation and apoptosis). Automated behavioural analysis technologies allow medium-to-high-throughput assessments.

Part of behavioural disruptions resulting from chemical exposure may rely on direct neurotoxicity or parental transfer, but beyond this direct relationship, behaviour is an interesting endpoint because it may also be an integrative indicator of several other physiological issues, e.g. metabolism, sensory organs, morphology or molecular pathways alteration.

### Behavioural test with laboratory fish species

The fact that behavioural responses may be integrative indicators of many physiological issues explains the rapid expansion of behavioural studies in ecotoxicological research during the last decades, particularly those using fish early life stages (ELS). Indeed, fish ELS are very amenable to high-throughput monitoring evaluation in multi-well plates. With that system behaviour like basal activity, response to a light change or other stimuli (indicative of anxiety) can be assessed. For the photo-motor response test with zebrafish embryos, it has been even shown that different compound groups induce different behavioural profiles which mean the test can be used to identify neurotoxic MoAs of compounds [230]. In addition, the possibility of using behaviour as a complementary approach to common fish embryo toxicity (FET) tests has recently been explored, demonstrating a significantly higher sensitivity of behaviour EC50 compared to FET LC50 for some compounds [231]. ELS have, however, a limited behavioural repertoire which is partially linked to a lower level of nervous system maturation compared to older stages. For example, it appears that learning associated with classical and operant conditioning is not efficient in zebrafish before 3 weeks of age, which is roughly the end of the larval stage [232]. In addition, and partly related to this limitation, the predictability of late outcomes from behavioural disruption in ELS is not obvious. As an example diquat, one of the compounds tested by Kluver et al. showed no behavioural disruption in ELS while it induced hyperactivity in older larvae [233].

Juveniles or adults have a much larger behavioural repertoire and/or cognitive abilities, which can easily be divided in behavioural units and evaluated. Behavioural disruptions resulting from exposure to organic pollutants, including PAHs or PCBs have been characterised using long-term dietary exposures to environmental mixtures. An increase in psychological stress is a common trait observed relating to all examined pollutants [234]. In case of PAHs, a decrease in the neurotransmitter serotonin could be the driver for this behavioural change [235]. More recently, behavioural changes in the offspring of fish exposed to a mixture of PCBs and PBDEs showed that even if exposed parents displayed no change in behaviour, two offspring generations showed a significant increase in anxiety as adults while behaviour of larvae was modified in up to four offspring generations.

### Behavioural tests with non-fish species

Van der Geest et al. showed that changes in ventilation behaviour of fifth instar larvae of the caddisfly *Hydropsyche angustipennis* occurred at approximately 150 times lower copper concentrations than mortality of first instar larvae [229]. Avoidance behaviour of the amphipod *Corophium volutator* to contaminated sediments was 1000 times more sensitive than survival [236]. Chevalier et al. tested the effect of twelve compounds covering different toxic MoA on the swimming behaviour of daphnids and observed that most compounds induced an early and significant swimming speed increase at concentrations near or below the 10% effective concentration (48 h) of the acute immobilization test. A reduction in defence and orientation behaviour of rusty crayfish after exposure to nicotinoid pesticides below morphological effect concentrations was observed by Sohn et al. [237]. The clam avoidance behaviour (closing valve after trigger) is suggested as a fast and easy screening tool for neurotoxicants [238]. *Diamesa zernyi larvae* from the wild also showed altered swimming behaviour after exposure to contaminants at low effect concentrations [239]. These examples and numerous others all showed that organisms may exhibit altered behaviour at relatively low and therefore often environmentally relevant toxicant concentrations [240]. Behavioural responses to toxicant exposure can also be very fast, allowing organisms to avoid further exposure and subsequent bioaccumulation and toxicity. A wide array of such avoidance responses have been incorporated in ecotoxicity testing [28, 241], including the avoidance of contaminated soil by earthworms (*Eisenia fetida*) [242], feeding inhibition of mussels (*Corbicula fluminea*) [238], aversive swimming response to silver nanoparticles by the unicellular green alga *Chlamydomonas reinhardtii* [243] and by daphnids to twelve compounds covering different toxic MoA [240].

### Field studies

Field studies are the most relevant approach to evaluate disruption resulting from chemical exposure but have drawbacks since it is (currently) almost impossible to disentangle the consequences of chemical exposure from other stressors such as food deprivation or environmental changes other than chemicals. On the other hand, laboratory experiments generally using optimal conditions except for the chemical exposure may lead to an underestimation of these exposure effects. They allow, however, the establishment of a potential direct link between exposure and physiological effects, including behavioural disruption. Indeed, behavioural abilities condition individual survival and fitness and hence have consequences at the population level. In the environment, behaviour abilities are important, e.g. to find food, to escape predators, to find new territory or partners for mating. All these behaviours are complex and rely on simpler so-called behavioural units, which are of ultimate importance in behavioural ecology because they are what can be measured [244].

Subtle changes in animal behaviour may affect trophic interactions and ecosystem functioning. Langer-Jaesrich et al. reported that midge larvae (*Chironomus riparius*) exposed to chlorpyrifos, a neurotoxic insecticide, showed a decrease in burrowing behaviour, resulting in an increase in the feeding rate of zebrafish preying on these exposed chironomids [245]. However, when exposing predators and prey simultaneously, no significant differences in the feeding rate of zebrafish were observed, suggesting impairment in prey recognition of the exposed zebrafish. In a laboratory toxicity experiment Hunting et al. (2013) observed that endpoints representing ecosystem structure and functioning, like bacterial diversity, bacterial activity and decomposition, responded much more sensitively to copper exposure than survival of the isopod *Asellus aquaticus* [246]. Monitoring the behaviour of the isopods by measuring the redox potential of the first upper cm of the sediment revealed that, although the isopods did not suffer from mortality upon the copper exposure, they became completely inactive. The lack of locomotion and shredding activity of the isopods caused the ecosystem processes to cease.

Since behavioural responses can be very fast, they can also be employed in early warning systems, with manifold applications. These so-called online-biomonitoring systems are installed to monitor the water quality permanently, and if the animals show an abnormal behaviour, an alarm goes off. This allows, for instance, the cessation of water intake for drinking water preparation. One of the most widely applied online-biomonitoring system is the Multispecies Freshwater Biomonitor™, based on a non-optical recording principle, the quadrupole impedance technique for several aquatic and sediment in/vertebrate species. This device quantitatively records behavioural patterns in a fully automated way on a real-time basis [247, 248]. Another approach uses fluorescence emissions and can be used to measure chlorophyll and thereby monitor phytoplankton biomass [249]. Daphnia and fish behaviour can also be monitored using cameras [250]. The valve opening of clams can be monitored using attached electrodes [251]. Behaviour is a fast and sensitive endpoint to toxicant exposure, allowing e.g. avoidance behaviour to be incorporated in online-biomonitoring systems. Subtle changes in animal behaviour may affect trophic interactions and ecosystem functioning, underlining the importance of incorporating behavioural endpoints into ecotoxicity testing.

## Mixture toxicity

The last decades have seen steadily rising volumes and numbers of industrial chemicals. Many of these substances are released into the environment, and thus, contamination comprises a mixture of different chemicals, each posing different characteristics and potential toxic responses towards humans and wildlife. Moreover, the large number of untested industrial chemicals and transformation products hampers comprehensive risk assessment of complex real-world mixtures of toxic chemicals. Regularly, contaminated environmental samples contain high numbers of many different pollutants which makes it an analytical challenge, including workup, separation, detection and quantification. Therefore, it is also difficult to assess the myriad of putative mixture effects and unknown toxic effects of old, new, and emerging chemicals in biological systems [252]. Additionally, the concentrations of pollutants in environmental samples are diverse and most of the time site-specific. This will cause also different biological response depending not only on the presence of certain pollutants but also on their relative composition [253]. One practical implication of this in connection to contaminated sites is that risk assessments still mainly rely on chemical analysis. However, such analysis does not always provide any information regarding the interactions between chemicals, including their integrated toxicological effects on organisms. Particularly, under regular environmental conditions organisms are exposed to multiple chemicals associated with different risks and the underlying toxic responses may generate toxic effects at levels exceeding the combined effects of single compounds. Thus, it has been recommended that combination effects of the entire mixture of contaminants should be evaluated in risk assessments [254, 255]. Therefore, many different toxic effects need to be checked. The neurotoxic potential of environmental chemicals presents a serious threat since they can, for instance, alter the behaviour of organisms. However, guidelines for neurotoxicity assessment consider only mammals and birds, and, thus far, there is no regulatory framework for neurotoxicity assessment in aquatic systems. DNT, which evaluates effects on the developing nervous system of foetuses, larvae, and juveniles, has special relevance for environmental risk assessment due to the ecological importance of early stages for recruitment and maintenance of natural populations. Despite this, DNT testing is currently required for human health risk assessment only and involves labour-intensive, time consuming and expensive animal testing. Consequently, few chemicals have been tested for DNT, leaving the potential impacts of untested chemicals and their combined effects unknown [256, 257]. Particularly, many pesticides are well known to act as neurotoxic compounds and commonly occur as complex mixtures in aquatic systems but concentrations are often low. Populations and communities of different species, however, can be dramatically impacted by low concentrations of pesticides, e.g. [258, 259]. Moreover, it is well known that pesticide mixtures contain compounds with similar and dissimilar MoAs [260] that can cause either concentration additive, synergistic neurotoxicity as well as unpredicted mortality depending on the combinations of different pesticides [260–262]. As a consequence, for chemicals with the same MoA, it might be possible to estimate their combined neurotoxicity using common models. However, known neuroactive compounds detected in the environment, such as pesticides, pharmaceuticals and heavy metals, occur together with thousands of chemicals of unknown neurotoxic potential, with different MoAs, and often in low concentrations. Therefore, there is a great demand for the development of meaningful effect-based methods to be applied for the screening and investigation of the neurotoxicity of chemicals and their combined effect.

One approach to identify/prioritize toxic compounds in mixtures is using toxic pressure modelling. Toxic pressure is the probability that measured field concentrations will be above laboratory effect concentrations causing adverse effects to an ecosystem [263]. The toxic pressure gives an indication what species might be affected by environmental contaminations. To calculate the toxic pressure, chemical analyses of compounds are used together with corresponding laboratory data. The toxic pressure of a mixture is determined by quantifying the potentially affected fraction (PAF) using species sensitivity distribution modelling for each compound in the mixture. In the following, mixture modelling is applied to estimate the mixture toxic pressure [264]. This method can be used either to calculate water quality standards, to prevent adverse effects or to predict potential effects of the current exposure situation by calculating the multiple-substances potentially affected fraction (msPAF). The program to calculate the msPAF or rank substances based on their toxic contribution is freely available [265]. To apply this method for eco-neurotoxicity risk assessment, laboratory data for neurotoxic effects for a variety of species are needed.

## Challenges in chemical analysis of neurotoxic compounds in environmental samples

For describing the behaviour, fate and effects of neurotoxic substances in the environment, analytical methods providing sufficient selectivity and sensitivity are required to achieve limits of quantification in the ng/L and µg/kg range for water samples and biota, respectively.

The diversity in the chemical properties of neurotoxic substances requires a variety of analytical methods for their identification and quantification. Chromatographic separation techniques combined with mass spectrometric detection are largely used for determining organic trace substances. For the determination of neurotoxic substances in solids, advanced extraction techniques are usually required to separate the analytes from many organic matrix compounds prior to analysis.

For the determination of organic substances, liquid chromatography coupled to a mass analyser (LC–MS) is the most frequently used analytical method. Electrospray ionization (ESI) is a robust ionization technique for polar neurotoxic substances. The MS/MS technique is used to reduce the chemical background thus leading to better signal-to-noise ratios. In triple quadrupole mass spectrometers, the analyte ions are selected in the first quadrupole filter and fragmented in the second quadrupole (collision cell), whereas one or more produced fragment ions are selected and detected in the third quadrupole filter. This so-called multiple reaction monitoring (MRM) enables the determination of neurotoxic substances in water in the lower ng/L range without any preconcentration techniques. For the analysis of biota, however, the much higher sample complexity (matrix) requires an increase in the selectivity of the mass spectrometer. One possibility to reach this is to replace the third quadrupole filter by a high-resolution mass analyser (HRMS). The time-of-flight (TOF) or Orbitrap technology is used in such hybrid instruments. A further benefit of using HRMS is the detection of all ions which are generated in the ion source. Thus, the datasets can be screened for substances which were initially not anticipated (so-called non-target screening).

In gas chromatography with mass spectrometric detection (GC–MS), the separation of analyte and matrix compounds (via the gas phase) usually takes place in capillary columns. Electron ionization (EI)—i.e. the interaction of energetic electrons with gas phase molecules—leads to ionization and fragmentation of analyte molecules. The fragment ions are subsequently detected.

For the analysis of metals, the preferred analytical methods are atomic absorption spectroscopy (AAS) or inductively coupled plasma mass spectrometry (ICP-MS). For determination of metals in solids, chemical digestion (e.g. HNO_3_/H_2_O_2_) is required. If the binding form or oxidation state of the metal should be retained during sample preparation, a suitable type of chemical digestion has to be selected. In this case, the ICP-MS is coupled with a separation method (e.g. ion chromatography).

## EDA in eco-neurotoxicity

Tens of thousands of unknown chemicals and toxicants can be detected in environmental samples, but in many cases only a few chemicals are responsible for significant risks to the ecosystem [7]. The concentration of chemicals may be variable in time and space and may include known but also unexpected and unknown environmental pollutants [7]. Since a comprehensive analysis of the whole chemical universe is technically impossible, it is necessary to develop tools to reduce the complexity of environmental contamination and identify the key contributors to a specific effect [7].

**Fig. 3:**
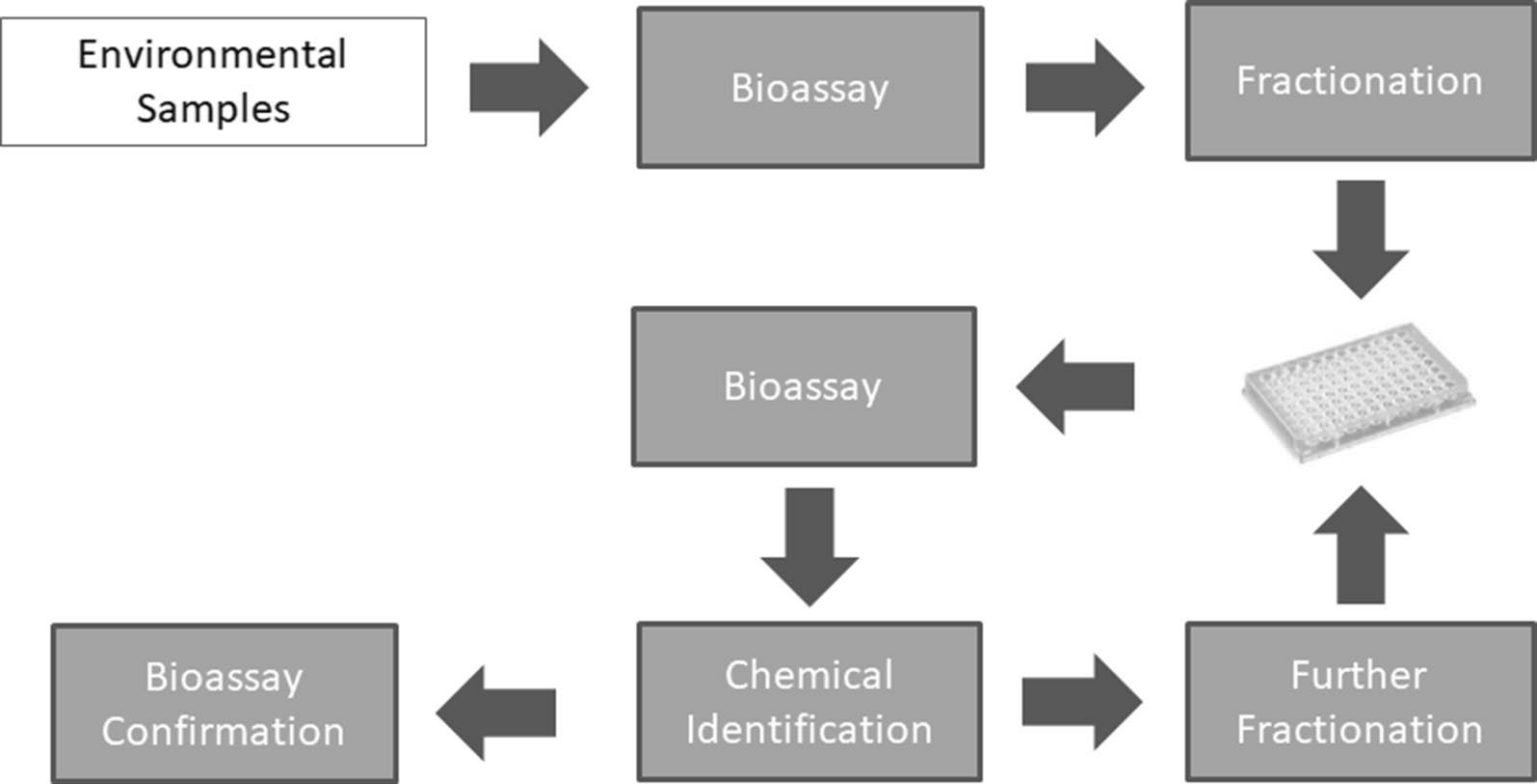
EDA workflow

Effect-directed analysis (EDA) was confirmed to be a powerful tool for the identification of chemicals causing effects to aquatic organisms or posing long-term risks such as endocrine disruption [266–268]. The EDA approach is based on the combination of chemical and biological tools isolating single substances or mixtures causing effects in laboratory bioassays. A typical EDA workflow (Fig. 3) follows certain steps starting with bio-testing of specific effects in an environmental sample. If the bio-testing reveals toxicity, the complexity of the sample can be reduced by fractionation, and fractions without biological activity are eliminated. Several fractionation steps can be performed until the toxic fractions are sufficiently isolated to allow identification by target and non-target chemical analysis [7].

Despite the fact that neurotoxicity was confirmed to be one of the most important MoAs observed in environmental samples [9], the number of EDA studies focused on the detection of neurotoxicity and neuroactive compounds is still very limited. One important example is give in the study of by Qu et al. who identified in vitro neurotoxicity induced by brominated flame retardants in environmental samples [269]. They used a combination of cell-based assays, fractionation and liquid/gas chromatography coupled with mass spectroscopy (LC and GC–MS) for the identification of neurotoxic brominated flame retardants in different environmental matrices [269].

In vitro AChE inhibition tests are most frequently used as effect-based tools in EDA for the identification of neurotoxic chemicals in water samples [233, 245, 270, 271]. The AChE assay was used for the identification of 50 neurotoxic compounds in spiked surface water samples by using an innovative 2-dimensional thin-layer chromatography [272]. Ouyang et al. developed a high-throughput EDA method for the fast identification of AChE inhibitors in wastewater treatment plant effluents by using LC–MS and a novel micro-fractionation workflow followed by an in vitro bioassay screening. This study successfully identified the presence of three potential AChE inhibitors (tiapride, amisulpride and lamotrigine) in environmental samples. It is important to underline that the in vitro AChE assay may still fail in the detection of causative neuroactive compounds during EDA studies and false-positive results are often observed in in vitro testing, since many molecules may form unspecific bindings with the purified enzymes [271].

In this context, in vivo and organism-level methods were also successfully applied in a few studies for the identification of neurotoxicity in environmental samples. In particular, behavioural and molecular tools using zebrafish embryos were used with good outcomes in different EDA studies for the identification of anticonvulsant drugs and natural toxins in plant extracts [273–275].

Finally, scientists are currently also working on the implementation of high-throughput techniques that are potentially useful in EDA for the identification of neurotoxic chemicals [276, 277]. As a major example, Fabel et al. successfully developed a fast novel workflow for the online biochemical detection after chromatography of potential AChE inhibitors [276].

## Cheminformatics approaches to assess eco-neurotoxicity

While many argue that assessing neurotoxicity requires animal models, due to the sheer number and potential mixtures of chemicals as well as animal testing requirements, a shift to in vitro assays and computational toxicity approaches is required [278]. Fortunately, large quantities of chemical biological data have been released into the public domain over the past 10 years. This includes data that have resulted from the US federal government-sponsored high-throughput screening programs such as Tox21 and ToxCast [279] testing thousands of chemicals against hundreds of assays (some of which are related to neurotoxicity). Furthermore, the development of online repositories such as ChEMBL [280] and PubChem [281] has resulted in collection and integration of chemogenomics data extracted from literature and/or directly deposited by researchers. Toxicological programs such as ToxCast (> 4000 chemicals and > 1000 high-throughput assays [282]) screening individual chemicals are extraordinarily resource intensive. Furthermore, as discussed above, a variety of neurotoxicity assays are needed to capture relevant endpoints [283]. Relevant neurotoxicity assays of varying sophistication and suitability for high-throughput studies include microelectrode arrays [128, 284], yeast assays [285, 286], assays using zebrafish [200, 287] and neural organoids derived from three-dimensional stem cell cultures [130].

In light of the chemical complexity in our environment, as discussed above, it is essential to connect predictive toxicity methods with the chemical signatures in samples to identify potential novel neurotoxicants in the environment before extensive neurotoxicity testing is performed. Computational toxicity approaches combined with literature-mining approaches and expert knowledge offer exciting ways to do this. Extensive work has already been performed by many research groups worldwide, screening drugs and potential neurotoxicants using various bioassays and collected into online resources such as ChEMBL and PubChem, including for instance the PubChem Classification Browser allowing sophisticated queries (e.g. results for Parkinson’s disease [288, 289]. In particular, the U.S. EPA National Center for Computational Toxicology provides their data for download [290], including their in vitro data, and is incrementally releasing their data and computational models through the CompTox Dashboard [291]. This provides access to chemistry data, integrates to in vitro assay and exposure data, and links to external resources and literature-mining functionality [292]. The Dashboard has both prediction and generalized read-across models (GenRA) [293]. This resource therefore provides direct access to a suite of data and tools that can support neurotoxicity research as a hub for data and information for chemicals, including e.g. lists of known (human) neurotoxins [294]. Initiatives such as the Abstract Sifter provide users with an interactive Excel sheet to explore the occurrence of chemicals and endpoints in the literature [295]. One approach to connect these computational efforts to environmental observations includes incorporating neurotoxicity endpoints into “virtual fractionation” investigations (i.e. correlating environmental signals with toxicological effects in a statistical approach before performing extensive EDA), which is possible on both known and unknown signals [7]. Incorporating toxicological information during identification efforts in chemical analysis can help to prioritize signals of interest (as performed for mutagenicity, e.g. [296] and is now possible in the in silico identification approach MetFrag [297]. Extensions to MetFrag [298] to better integrate the computational toxicity information offered by the CompTox Dashboard, PubChem and other resources are underway. This will offer exciting new ways to prioritize candidates to improve non-target screening efforts [29] in all ecotoxicity contexts, including neurotoxicity. Connecting these efforts to big data approaches such as those implemented in GNPS [299] will offer novel data-mining opportunities in the years to come [300].

## The AOP concept in eco-neurotoxicity

The adverse outcome pathway (AOP) framework is intended to structure evidence of mechanistic toxicity information for a more effective use in risk assessment and regulatory decision making [301]. An AOP is triggered by a molecular initiating event (MIE), which is the interaction of a compound with a molecular target such as a receptor, and further outlines the sequence of key events (KE) along different levels of biological organization from the molecular level up to the adverse outcome at a level of regulatory concern. The latter is generally the organismal level in human toxicology and the individual or population level in ecotoxicology. One of the earliest conceptual AOPs proposed concerned the neurotoxicity of domoic acid [302]. The OECD has taken on the task of coordinating international collaboration and harmonization of AOP development. The AOP-Wiki [303] aims at centralizing AOP descriptions into a publicly available repository to facilitate their application by the (eco)toxicologist community from industry, academia and regulatory institutions.

Two AOPs for neurotoxicity have been endorsed by the OECD, i.e. “Chronic binding of antagonists to *N*-methyl-d-aspartate receptors (NMDAR) during brain development inducing impairment of learning and memory abilities” [304] and “Binding of agonists to ionotropic glutamate receptors in adult brain causing excitotoxicity that mediates neuronal cell death, contributing to learning and memory impairment” [305, 307]. Four more neurotoxicity AOPs are currently either under review or approved (AOPs 10, 12, 42, 54). These describe binding to ionotropic GABA receptors leading to epileptic seizures [306], the role of NMDAR antagonists in neurodegeneration during ageing [307], and neurodevelopmental toxicity through interference with thyroid hormone synthesis (2 AOPs, [308]). For all of these AOPs, the focus is mostly on human health, although in some cases fruit fly, zebrafish or bobwhite quail data are included as weight of evidence. About 25 other AOPs currently being developed in the AOP-Wiki with a primary human toxicological perspective mainly focus on thyroid hormone disruption, and adrenergic, dopaminergic, serotonergic and opioid pathways.

**Fig. 4:**
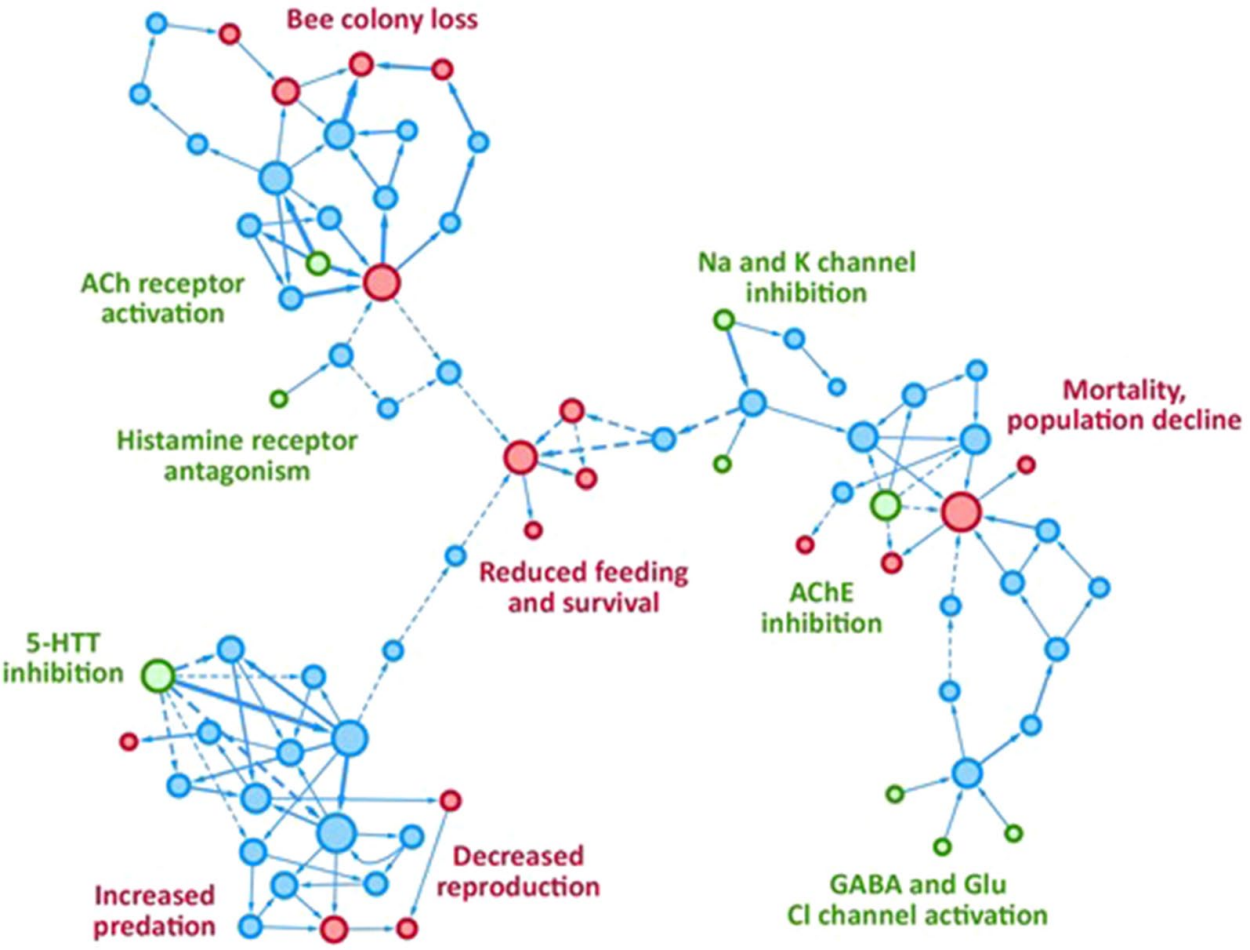
AOP network showing all AOPs relevant to eco-neurotoxicity that are available in the AOP-Wiki (https://aopwiki.org; Accession date: April 30, 2018; AOP numbers 16, 77, 78, 79, 80, 82, 87, 88, 89, 90, 91, 93, 94, 95, 97, 98, 99, 113, 160, 161, 178, 195, 197, 203, 204; Network constructed using Cytoscape 3.6.1). Nodes in the network are key events (KEs), and edges represent key event relationships (KERs). Molecular initiating events are displayed in green, adverse outcomes in red. All other KEs are depicted in blue. Solid edges are adjacent KERs, dashed arrows are KERs that have been defined as non-adjacent (see the OECD’s users’ handbook for developing and assessing AOPs for more information, [333]). Node size represents node degree (the total number of KERs connecting the KE to the network, Villeneuve et al., and edge thickness represents the number of times a given KER is part of constituent AOPs in the network. While some of the AOPs in the network have been published, many are in early stages of development, and none have been reviewed or endorsed by the OECD. This figure and its annotations therefore merely illustrate the current focus areas of eco-neurotoxicity AOP developers in the AOP-Wiki, as well as the interrelatedness of these research topics. The AOP network does not make any inference about the scientific validity of the underlying AOPs, nor can it at this stage be used for in-depth biological interpretation or regulatory application. ACh, acetylcholine; AChE, acetylcholinesterase; 5-HTT, 5-hydroxytryptamine (serotonin) transporter; GABA, gamma-aminobutyric acid; Glu, glutamate; Na, sodium; K, potassium; Cl, chloride

AOPs covering neurotoxicity in an ecotoxicological context (about 25 in total in the AOP-Wiki) are at this point in time less advanced in general. Development of eco-neurotoxicity AOPs currently focuses on interference with cholinergic pathways leading to colony loss in bees and acute mortality in multiple species [309, 310], serotonergic (5-hydroxytryptamine transporter, 5-HTT) pathways leading to population changes in bivalves [311], and histamine, glutamate and GABA pathways, while some well-characterized pathways in mammals are currently lacking. Figure 4 shows an AOP network illustrating the current focus areas of eco-neurotoxicity AOP developers in the AOP-Wiki, as well as the interrelatedness of these research topics.

**Fig. 5:**
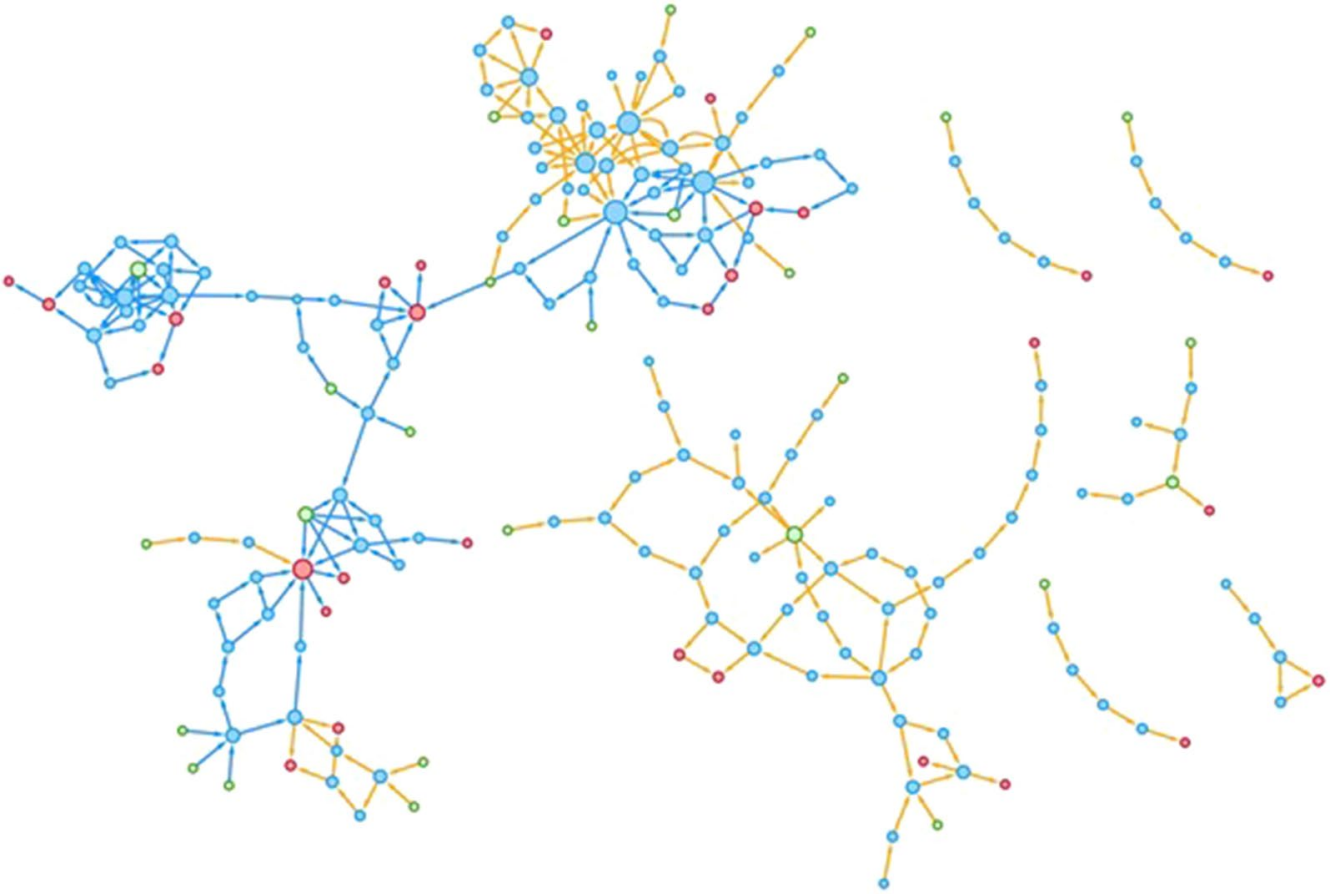
Assembly of AOP networks showing all AOPs relevant to human (orange) and eco-neurotoxicity (blue) that are available in the AOP-Wiki (https://aopwiki.org; Accession date: April 30, 2018; Eco-neurotoxicity AOP numbers 16, 77, 78, 79, 80, 82, 87, 88, 89, 90, 91, 93, 94, 95, 97, 98, 99, 113, 160, 161, 178, 195, 197, 203, 204; Human neurotoxicity AOP numbers 3, 8, 10, 12, 13, 17, 26, 42, 48, 54, 73, 104, 112, 126, 134, 152, 164, 170, 214, 215, 221, 222, 223, 224, 225, 226, 230, 231, 233, 234, 235, 236; Network constructed using Cytoscape 3.6.1). Nodes in the network are key events (KEs), and edges represent key event relationships (KERs). Molecular initiating events are displayed in green, adverse outcomes in red. All other KEs are depicted in blue. Node size represents node degree (the total number of KERs connecting the KE to the network, [334]). KERs in blue are part of eco-neurotoxicity AOPs and correspond to Fig. 4, KERs in orange are part of human neurotoxicity AOPs. The AOP network does not make any inference about the scientific validity of the underlying AOPs, nor can it at this stage be used for in-depth biological interpretation or regulatory application

Currently, AOPs are mostly being developed separately for human toxicology and ecotoxicology, while many mechanisms are shared, especially among vertebrate taxa. The philosophy of modular AOP development facilitated by the AOP-Wiki, the evaluation of taxonomic applicability of AOPs and the concept of AOP networks [312], has the potential to aid in bridging the gap between these two fields. Figure 5 shows that although development of many human and eco-neurotoxicity AOPs are currently rather distinct processes, some connections between both fields are being established. The AOP of AChE leading to acute mortality provides a good example, since a specific effort has been made to make a broad assessment of taxonomic applicability based on species sensitivity distributions and sequence similarity assessment of the molecular target [310]. Other examples of studies that have attempted to broaden the taxonomic applicability domain of neurotoxicity AOPs include Gong et al. [306] for ionotropic GABA receptor antagonism and Fay et al. [311] who used Sequence Alignment to Predict Across Species Susceptibility (SeqAPASS, [313]) to assess conservation of the molecular target, in this case 5-HTT, across species.

## Molecular targets of environmental contaminants with neurotoxic/neuroactive mode of action

AOPs for eco-neurotoxicity would support the development of bioassays for the detection of (converging) KEs that can be used to evaluate complex mixtures of chemicals triggering diverse neurotoxic AOPs. The recent AOP-Wiki (including putative AOPs) already reflects various targets and associated molecular initiating events that are known to lead to interference with the function and development of the nervous system (see previous section). Furthermore, many environmental contaminants are known to interact with a biological target relevant for the function of the nervous system. This interaction can mostly be inferred from the intended biological effect in case of pharmaceuticals, pesticides and biocides. However, it remains to be demonstrated whether the intended biological target, such as the serotonin receptor, is also the MIE leading to a relevant adverse outcome and if other mechanisms of action than the reported pharmacological or insecticidal mechanism are leading to a neurotoxic/neuroactive effect as well. Compounds that have not been designed for biological activity (such as industrial chemicals) could also unintendedly impact on a target relevant for neuroactivity. Furthermore, available information on neuroactivity/neurotoxicity often stems from a specific animal class (e.g. mammals in case of pharmaceuticals) and it is not known whether the neuroactive mechanism also applies to other organisms.

An estimation of whether the recent AOP-Wiki captures some of the reported biological targets and associated AOPs of environmental contaminants (based on available information on neuroactivity) can be made by analysis of the mechanisms of action of contaminants frequently detected in the environment by large-scale analytical chemistry. By combining the data from studies that measured the concentration of several hundred different chemicals in three European river catchments (Danube, Rhine, Mulde/Saale), compounds with neurotoxicity/neuroactivity to any species were found as the largest group of chemicals with known MoA or target accounting for 13% of the 426 chemicals that were detected in at least one of the catchments [9].

By assigning a major molecular target to each of the neurotoxic/neuroactive chemicals in the study of Busch et al. using databases (DrugBank, IRAC) and public literature, 16 different mechanisms were identified (Table 2). Based on the hazard quotient (HQ), i.e. the ratio of observed concentrations and the measured or predicted effect concentrations for fish and daphnids, the different mechanisms of neuroactivity can be ranked with respect to their environmental relevance. Fifteen of those refer to a functional interference and only one represented a compound with developmental neurotoxicity (simazine, a herbicide with evidence to inhibit proliferation and differentiation of dopaminergic nerve cells [314]). This assignment could be partially biased since for some chemicals (i) the mechanism is not precisely known, (ii) other (neuroactive) mechanisms are reported as well and/or (iii) the data often stem from drug development or drug toxicity studies, and information whether the mechanism is applicable to environmental organisms is lacking. Nevertheless, the HQ assessment represents a useful approach to prioritize neurotoxicity AOPs that would be of major interest in ecotoxicology.

**Table 2:** Compounds with neuroactive mode of action that were found during large-scale chemical analysis of samples from three European river basins (Danube, Rhine, Mulde/Saale)

MIE	Compound name	HQ histogram fish	HQ histogram daphnia	Captured in AOP-wiki?	Reported species specificity
All	n/a			n/a	n/a
Acetylcholinesterase inhibition	Triphenylphosphate, rivastigmine, tris(1,3-dichloroisopropyl)phosphate (TDCPP), carbofuran, carbetamide, methomyl, methiocarb, pirimicarb, ethyl-azinphos, chlorpyrifos, diazinon, chlorfenvinphos, chlorpyrifos-methyl, dimethoate, carbaryl, TMPP (tris (methyl phenyl) phosphate)			Yes	Conserved among vertebrates and invertebrates [298]
GABA receptor antagonism	Fipronil			Similar (non-competitive GABA receptor blocking)	Compound-specific differences in target sensitivity [306]
Serotonin reuptake inhibition, leading to stimulation of serotonergic neurons	*N*,*O*-didesmethyl venlafaxine, citalopram, *O*-desmethylvenlafaxine, venlafaxine			Yes	Human pharmaceuticals but potential neuroendocrine effects in invertebrates [307]
Antagonism of serotonin, dopamine and/or alpha-adrenoreceptors	Metoclopramide, trimipramine			Not available	
Opioid receptor agonism	Tramadol, morphine, codeine, methadone			Yes	
Nicotinic acetylcholine receptor (nAChR) agonism	Thiacloprid, acetamiprid, imidacloprid, levamisol, thiamethoxam, clothianidin, cotinine			Yes	Specific for Insects,in contrast to parent compound nicotine [308])
Enhancement of GABA action via allosteric binding to GABA receptor	Primidone, flunitrazepam, diazepam, midazolam, oxazepam, pentobarbital, lorazepam			Not available	
NMDA-type glutamate receptor antagonism	Dextromethorphan, ketamine, 1-adamantylamine			(only for agonism)	
Inhibition of adenosine receptor (CNS stimulation)	Clopidogrel (and derivatives), caffeine			Not available	
Dopamine reuptake inhibition, leading to stimulation of dopaminergic neurons	Bupropion, cocaine			Not available	
Dopamine receptor antagonism	Sulpiride, amisulpride			Not available	
Voltage gated sodium channel antagonism	Lidocaine, carbamazepine (including derivatives), lamotrigine			Yes	
Release of serotonin, dopamine and noradrenaline	MDMA/methylendioxymethamphetamine, MDEA/3,4-methylenedioxyethamphetamine			Not available	
Proliferation and differentiation of dopaminergic nerve cells	Simazine^a^			Not available	
Inhibition of monoamine oxidase (leading to reduced metabolism and hence, increased levels of serotonin and noradrenaline)	Moclobemide			Not available	
Voltage gated calcium channel inhibition (reduces release of transmitters such as glutamate or noradrenaline)	Pregabalin, gabapentin, gabapentin-lactam			Not available	

Compounds were grouped according to a common target and molecular initiating event (MIE). Data were extracted from Busch et al. and supplemented with more details on the major reported mechanism of action. Note that the mechanism of action can be highly species-specific but that species specificity is often not known. Hazard quotients (HQ = measured environmental concentration/effect concentration) represent predicted values (baseline toxicity multiplied by a factor of 10 or 100 for non-narcotic compounds) if no measured effect concentrations were available (please refer to Busch et al. for further details). Higher log HQs (close to zero) indicate that environmental concentrations are likely to cause a biological effect. Note that all AOPs of the AOP-Wiki (http://www.aopwiki.org) are under development

^a^Simazine is a herbicide, but strong evidence for a neurotoxic mode of action in non-target organisms was reported [304]

The HQ analysis using data from the study of Busch et al. [9] indicated that AChE inhibition is one of the most dominant MIEs, represented by 16 of the detected neurotoxic chemicals and displaying the highest HQs. These compounds are mainly pesticides but also include a few drugs such as rivastigmine used in the treatment of Alzheimer disease [315]. An elaborated AOP is available for fish toxicity [310] and assays that relate to the MIE (AChE enzyme activity, e.g. [316]) or key events (e.g. behaviour analysis in fish embryos relating to the KE of hyperactivity and paralysis, [231]) are available. AChE inhibitors are also well known for their relatively weak species specificity and they affect mammals and invertebrates as well. The highest HQs were found for invertebrates, which probably reflect the high sensitivity of invertebrates (AChE inhibitors have been mainly developed as insecticides). The ranking of AOPs for neuroactivity/neurotoxicity in ecotoxicity could be used to prioritize further development of AOPs by applying the following principles: (i) establishing AOPs for MIEs targeted by compounds with high HQ, (ii) filling of data gaps to address species specificity, (iii) establishing new AOPs with focus on organisms and KE-related assays that provide the opportunity to develop screening assays, and (iv) identification of converging KEs that could be used to develop bioassays that would allow to detect compounds targeting different AOPs. Identification of converging KEs would be useful in case of screening of compounds or the assessment of the cumulative impact of complex environmental mixtures using bioassays, given that otherwise large testing batteries to address different mechanisms would be required.

## Development of neurobehavior AOPs to predict population level impacts across species

Behaviour assays can be powerful endpoints for contaminant experiments because behaviour integrates the internal physiological state of an animal with the animal’s response to external stimuli at the same time [317] and can be a good indicator and measure of sublethal effects. Changes in behaviour can be incorporated into the AOP framework as whole organism responses, and it is useful to do so because of the potential utility of using AOPs for cross-species extrapolations and ecological risk assessment, and for using the fish embryo as a substitute for many traditional toxicity tests. Neurobehavioural impacts of contaminants have been documented in previous laboratory [318, 319] and/or field [320] studies involving fish.

Embedding behaviour into an AOP that predicts ecologically relevant adverse outcome is challenging because it is difficult to interpret subtle changes in behaviour in terms of population demographics. There have been some development in methods to link behavioural outcomes to population relevant impacts that are suitable for ecological risk assessment [321–324]. As fish larvae are particularly sensitive to predation and starvation, impaired behaviour related to foraging and predator avoidance may have drastic consequences to the individual [325]. Ecologically relevant behaviours that should be measured in contaminant-exposed fish include swimming speed, startle response, reactive distances, prey capture ability, learning and memory. These behaviours can be related to the probability of escaping a predator, capturing prey for feeding and encounter rates using statistical models. Some examples of directed laboratory studies that were used to focus on behaviour relevant to ecological processes such as foraging and predator avoidance and subsequently used to construct an individual based model calibrated for particular species and populations are available [323, 324].

With behaviour as a pivot key event in the development of an AOP, there are challenges associated with incorporating suborganismal information. Prior to the onset of clinically apparent neurobehavioural damage, significant changes in brain neurochemistry occur and monitoring such changes using neurochemical biomarkers or key events represents an objective and early means to identify key molecular initiating events. The associations between measurable disruptions in brain gene expression measured using RNAseq, metabolomics and reverse engineering, and probabilities of capturing prey and avoiding predators can then be incorporated into a larval fish cohort model (e.g. [321, 322, 324, 326]), to predict survival and growth of the cohort. Cohort survival and growth can also be easily translated into parameters relevant to common metrics used by regulatory agencies (e.g. EC50; [322]), and can also be incorporated into a matrix population model to predict population effects of contaminant via induced changes in behaviour [327]. However, more work is needed to use valuable behavioural assays to create powerful AOPs that link the effects of chemicals from the level of molecular initiation all the way to the population.

The AOP framework has been proposed to be suitable for cross-species extrapolations [328]; however, for neurobehavioural studies, there are distinct challenges that will have to be addressed. Right now, in ecological risk assessment just a few selected species that are easy to maintain in the laboratory are used to make decisions on over 32,500 species of fish [328]. Test species are rarely selected for geographical range, physiology or life history. However, as baseline behaviour can vary widely between species, and as it is challenging to get non-laboratory model organisms to behave normally in laboratory settings, applying the neurobehavioural response as a key event to indicate population outcomes can be difficult for many species [326]. To perform cross-species extrapolations, the AOP framework can be used to identify key events that are more informative and easier to measure. For example, it may be possible to develop a neurobehavioural AOP in a model species like the zebrafish and extrapolate to other species of ecological importance. A neurobehavior AOP that targets larval life stages (a stage that is easy to maintain and test in the laboratory) would be ideal for cross-species extrapolations and would be in direct concordance with the spirit of the NRC 2007 report “Toxicity Testing in the 21st Century: A Vision and a Strategy” [329]. To use the zebrafish or another model species to predict neurobehavior adverse outcomes in another species, the same information should be collected at every level of biological organization across multiple KEs to determine what information is useful for cross-species comparisons, what information is lost by using a model species and the errors associated with cross-species assumptions [326, 330]. Essentially, there may be KEs that can be measured at the suborganism level that are better at predicting population outcomes than neurobehavioural KEs. Once a few cross-species comparisons have been completed using the AOP framework in a systematic way, the neurobehavioural KE may be replaced with a molecular or cellular KE and used to make population predictions for alternate species instead. These molecular/cellular KEs are likely to be more amenable to high-throughput methods than behavioural responses.

## Conclusions

### Current situation

Considering the increasing numbers of environmental contaminants, their unknown neurotoxic potential, the physiological and morphological complexity of the nervous system, and the wide range of potential consequences of neurotoxicity, it is a major challenge to identify and advance neurotoxicity testing strategies and methods that improve neurotoxicity and eco-neurotoxicity assessment [331]. Additionally, neurotoxicity end especially eco-neurotoxicity is a challenging endpoint. Developmental processes, hormones, epigenetic regulations, the microbiome and many other processes can all impact nervous system functioning. Toxic MoA can be rather simplistic like AChE inhibition, but even then, differences in species sensitivities can make it rather difficult to assess eco-neurotoxicity.

### Recommendations

#### Effects

Many different compounds such as flame retardants, plasticizers, pesticides and pharmaceuticals are suspected to be neurotoxic based on epidemiological studies in humans and eco-neurotoxic based on environmental species. Neurotoxic effects can be seen after exposure to trace levels of micropollutants. There is only very limited information available when it comes to eco-neurotoxic effects but based on what is known we propose that:

- Neurotoxicity studies should include the sensitive stages of development for the selected test species. Additional testing for developmental effects, including long-term effects, with, e.g. zebrafish, medaka, bird eggs, should be performed.
- Epigenetic effects should be investigated. Epigenetic effects can indicate multi-generation effects, which are especially relevant when looking at whole ecosystems.
- The biomolecular process like transcriptomic or metabolomic changes and their link to behavioural alterations should be investigated in more detail.
- Due to the close link between the hormonal system and the nervous system, endocrine effects should be tested for potential neurotoxic effects.
- Most known neurotoxic MoA describes interference with synapse functioning like binding to neurotransmitter receptors or effects on calcium homeostasis. Species-specific differences in sensitivity are often due to differences in the detoxification metabolism. This should be taken under consideration when selecting species or assays. To compensate for this, pre-metabolization steps could be included or metabolites tested as well as parent compounds.
- The application of TK–TD models might be helpful to better understand the impact of different exposure scenarios.
- Mixture assessments for solely neurotoxic substances but also in combination with non-neurotoxic substances are needed.

#### Chemistry

The number of compounds in daily use and consequently emitted in the environment is increasing and monitoring all of them in the environment is challenging, especially in biota. Potential solutions are to

- Identify potential unknown neurotoxicants within an environment using effect-based monitoring that can be combined with effect-directed analysis. Reliable, high-throughput assays are needed to enable this approach.
- Use cheminformatics approaches or toxic pressure modelling to incorporate computational toxicological information with chemical (monitoring) data.

#### Assays

Current in vivo test systems are mostly developed for human neurotoxicity assessment, although they could be easily adapted for ecological use. The current systems mostly do not allow large-scale screening. To be able to screen all relevant environmental contaminants for their neurotoxic potential, novel screening approaches are needed.

These assays should be:

- Applicable for high-throughput, cost-efficient and alternatives to animal testing.
- Due to the complexity of the nervous system, only a test battery of in vitro assays covering different MoAs will be able to replace current whole animal tests. To develop such a battery, it is necessary to understand the involved mechanisms of toxicity and to establish causal relationships between neurotoxicity endpoints and behavioural consequences [332]. AOPs might help in selecting targets.
- Developing a set of test chemicals, covering different MoAs might be useful for assay validation studies.

#### Risk assessment

In Europe, neurotoxicity of compounds is only assessed for human exposure thus far, and only for medium and high production volume chemicals, not based on exposure levels. Effects on organisms in the environment are not considered.

- Online-biomonitoring systems for measuring behaviour of sensitive organisms within an ecosystem could be used as early warning systems.
- EDA and Toxic Pressure modelling could be used to identify hazardous substances.
- To include eco-neurotoxicity assessment, cheminformatics in combination with in vitro screening tests could be used as *first tier* to screen compounds found in an ecosystem for neurotoxicity. AOPs can be helpful in guiding this process. In vitro assays for relevant mechanisms of neurotoxicity could then be implemented, validated and combined in a test battery. For eco-neurotoxicity assessment, species differences should be considered in the selection of in vitro assays.
- In a *second tier, a* test battery using small species covering different trophic levels could be used to assess risks of ecosystems. Such testing batteries could be also applied for mixture assessment and in combination with effect-directed analysis to identify neurotoxic pollutants in the environment.

There is a clear need to better understand how compounds can cause eco-neurotoxicity and how this differs between species. Only when the most sensitive species is protected, harm to the environment can be prevented. Novel methods or strategies need to be developed, able to deal with the large amounts of environmental pollutants, the complexity of the nervous system and the diversity of the ecosystem. Such methods will allow to implement eco-neurotoxicity as part of EU legislation.

## Abbreviations

AA-EQS: annual average environmental quality standards
AAS: atomic absorption spectroscopy
AChE: acetylcholinesterase
AOP: adverse outcome pathway
CNS: central nervous system
DNMT: DNA methyltransferase
DNT: developmental neurotoxicity
ECHA: European Chemicals Agency
EDA: effect-directed analysis
EDC: endocrine disrupting chemical
EFSA: European Food Safety Agency
ELS: early life stage
ESI: electrospray ionization
FET: fish embryotoxicity test
GABA: g-aminobutyric acid
GC–MS: gas chromatography coupled to mass spectrometry
GenRA: generalized read-across models
HQ: hazard quotient
HRIV: health-related indicator value
HRMS: high-resolution mass analyser
ICP-MS: inductively couples plasma mass spectrometry
KE: key event
LC–MS: liquid chromatography coupled to mass spectrometry
MAC-EQS: maximum allowable environmental quality standards
MEA: multielectrode array
MIE: molecular initiating event
MoA: mode of action
MRM: multiple reaction monitoring
msPAF: multiple-substances potentially affected fraction
NMDAR: *N*-methyl-d-aspartate receptors
PAF: potentially affected fraction
REACH: registration, evaluation, authorisation and restriction of chemicals
SIMONI: smart Integrated monitoring
TEI: transgenerational epigenetic inheritance
TK–TD: toxicokinetic–toxicodynamic
TOF: time-of-flight
WFD: Water Framework Directive
